# The impact of emotional dysregulation and comorbid depressive symptoms on clinical features, brain arousal, and treatment response in adults with ADHD

**DOI:** 10.3389/fpsyt.2023.1294314

**Published:** 2024-01-05

**Authors:** Jue Huang, Nicole Mauche, Eike Ahlers, Holger Bogatsch, Pierre Böhme, Thomas Ethofer, Andreas J Fallgatter, Jürgen Gallinat, Ulrich Hegerl, Isabella Heuser, Knut Hoffmann, Sarah Kittel-Schneider, Andreas Reif, Daniel Schöttle, Stefan Unterecker, Maria Strauß

**Affiliations:** ^1^Department of Psychiatry and Psychotherapy, University of Leipzig, Leipzig, Germany; ^2^Department of Psychiatry and Psychotherapy, Charité – Universitätsmedizin Berlin, Berlin, Germany; ^3^Clinical Trial Centre Leipzig, Faculty of Medicine, University of Leipzig, Leipzig, Germany; ^4^Department of Psychiatry Psychotherapy and Preventive Medicine, University Hospital of Bochum, Bochum, Germany; ^5^Department of Biomedical Magnetic Resonance, University Hospital of Tübingen, Tübingen, Germany; ^6^Department of Psychiatry and Psychotherapy, University Hospital of Tübingen, Tübingen, Germany; ^7^Tübingen Center for Mental Health (TüCMH), University Hospital of Tübingen, Tübingen, Germany; ^8^Department of Psychiatry and Psychotherapy, University Medical Center Hamburg-Eppendorf, Hamburg, Germany; ^9^Department of Psychiatry, Psychotherapy and Psychosomatic Medicine, University Hospital of Frankfurt – Goethe University, Frankfurt am Main, Germany; ^10^Department of Psychiatry, College Cork, Cork, Ireland; ^11^Department of Psychiatry, Psychosomatics and Psychotherapy, University Hospital of Würzburg, Würzburg, Germany

**Keywords:** ADHD, emotional dysregulation, EEG, brain arousal regulation, adult, methylphenidate

## Abstract

**Introduction:**

The role of emotional dysregulation (ED) in attention-deficit/hyperactivity disorder (ADHD) has become an important issue. This study, in which we analyzed data from a predictive pharmaco-EEG-trial, aimed to examine whether symptoms of ED in adult ADHD affect ADHD symptom severity, brain arousal regulation as measured by resting EEG, and the response to stimulant medication.

**Methods:**

ED is defined as having a sex- and age-corrected *T*-score of >70 on the emotional lability subscale of the German version of Conners’ Adult ADHD Rating Scale. A total of 115 participants were included in the study, 56 of whom had ED. Participants with ED were more impaired in terms of the severity of core ADHD symptoms, especially inattentive symptoms, comorbid depressive symptoms, interpersonal relationships, and quality of life. In addition, participants with ED were more likely to report a total score above 13 on the Beck Depression Inventory-II, which was considered to be the cutoff for mild depression.

**Results:**

No differences were found between the ED and non-ED groups in response to stimulant medication or in brain arousal regulation. In addition, there was no significant effect of ED with comorbid depressive symptoms on treatment response. There was a trend for subgroups that showed a change in brain arousal regulation associated with symptom improvement.

**Discussion:**

Our findings may support the assumption that ED may be an important feature of ADHD. The use of EEG-based brain arousal regulation as a diagnostic and predictive tool in ADHD in the presence of ED and comorbid depressive symptoms should be further investigated.

## 1 Introduction

Attention-deficit/hyperactivity disorder (ADHD) is a common childhood-onset neurodevelopmental disorder with a global prevalence of 2–7% in children/adolescents (1). Estimates of the persistence of ADHD into adulthood vary widely, from 40 to 77% (2–6), resulting in approximately 3% of adults in the population being affected (7). ADHD is characterized by cross-situational impairments in attention, hyperactivity, and/or impulsivity, describing three distinct subtypes (8), and results in detrimental impacts on social, financial, and professional functioning (9).

To date, the diagnosis of ADHD is primarily based on clinical presentation and functional impairment assessed through interviews and self-report questionnaires. The recommended multimodal treatment of ADHD in adults includes pharmacological and non-pharmacological therapies, with psychostimulants being the first line of treatment (10). Although there are several effective medications for the treatment of ADHD, efficacy is highly variable (11), and diagnosis and treatment success are only measurable at the psychopathological level. An objective and reliable parameter with a prognostic value for pre-post-treatment comparison is still missing.

Electroencephalography (EEG) has been frequently used in the clinical context of diagnosis and treatment (12). Some atypical patterns of neural activity have been associated with ADHD (13). However, recent reviews have concluded that EEG biomarkers are not yet reliable enough to be used for diagnostic purposes but could be a diagnostic adjunct (14, 15). The most consistent markers of ADHD in the resting EEG are elevated levels of absolute and relative theta power and increased alpha activity (15). In addition, Bellato et al. suggested in a systematic review (16) a potential link between difficulties in regulating states of wakefulness and deficits in attention and executive function as a core problem associated with ADHD (17).

In line with this, the brain arousal regulation model proposes that difficulties in regulating arousal are an important factor in the pathology of ADHD. Hyperactivity and sensation seeking can be interpreted as an autoregulatory response to an unstable level of vigilance in the sense of brain arousal (18), and numerous findings seem to support this hypothesis (e.g., 16, 19). Different levels of brain arousal (termed as EEG-vigilance stages) and its regulation (indexed by arousal stability scores) can be measured by taking into account the frequency patterns and low-resolution electromagnetic tomography (LORETA)-based cortical distribution of EEG activity. The Vigilance Algorithm Leipzig (VIGALL 2.1), a free downloadable EEG-based algorithm (https://www.deutsche-depressionshilfe.de/forschungszentrum/aktuellestudien/vigall-vigilance-algorithm-leipzig-2-1 assessed on September 12 2023), has been developed and validated for this purpose by our group (19–24).

In a previous open-label, single-arm, multicenter confirmatory study in adult ADHD (aADHD) patients, we investigated whether the stability of brain arousal regulation, as measured by VIGALL 2.1, has a prognostic value in predicting response to methylphenidate therapy (25). We hypothesized first that unstable arousal regulation prior to medication would have a prognostic value in predicting response to methylphenidate treatment, and second, that the level of brain arousal would be associated with symptom severity, as assessed by the German version of the Conners’ Adult ADHD Rating Scale [CAARS; (26)]. We could not confirm our hypothesis; in contrast to our previous studies, the results strongly suggest that brain arousal regulation was not meaningfully associated with treatment response in this population. Possible explanations include the severity of ADHD symptoms and the high proportion of participants with comorbid depressive symptoms. Approximately 22% of the patients achieved very high CAARS scores (*T*-score of 90) at baseline, and more than a third suffered from comorbid depressive symptoms, which were positively correlated with hyperstable brain arousal regulation. It was also shown that patients with unstable brain arousal regulation, and no relevant comorbid depressive symptoms had the largest therapeutic success although this was not significant. Our findings support the generally accepted fact, that at least a subgroup of patients with aADHD are hypoaroused. In summary, and with regard to the results of our previous studies, it can be assumed that the results in this population may have been influenced by other variables.

Emotional dysregulation (ED) in ADHD has become an important issue. According to the Diagnostic and Statistical Manual of Mental Disorders (DSM-5), ED is recognized as a diagnostic feature to support the diagnosis of ADHD. Research shows that ED occurs in up to 70% of people with aADHD (27–29). Emotional regulation (ER) is generally understood as an individual’s ability to regulate extrinsic and intrinsic processes in order to achieve one’s goal (30), and ED is defined as emotional experiences or expressions that interfere with goal appraisals. Previous theorists have described four patterns that tend to characterize individuals who are emotionally dysregulated. These include ineffective enduring of emotions and regulatory attempts, emotions interfering with appropriate behavior, inappropriate emotional expression and experience in context, and too abrupt or too slow changes in emotions (29, 31, 32). In this study, we focused on the clinical expression of ED in individuals with ADHD, specifically described as hot temper, tantrums, or irritability. We measured this using the impulsivity/emotional lability subscale of the CAARS. This subscale was designed to measure the clinical symptomatology as impulsivity and emotional lability, including the clinical expressions of ED mentioned above. In order to distinguish impulsivity from emotional dysregulation in this study, impulsivity is considered as the tendency to act on impulse without adequate forethought (33), and impulsivity often results in mistimed and premature actions and can occur without any pre-existing emotional arousal. The six items included in this subscale measure emotional lability: “I am easily frustrated,” “I have a short fuse/hot temper,” “I still throw tantrums,” “Many things set me off easily,” “My moods are unpredictable,” and “I am irritable”; the remaining six items were designed to measure impulsivity: “I blurt things out,” “I say things without thinking,” “I interrupt people when they are talking,” “I make comments/remarks that I wish I could take back,” “I step on people’s toes without meaning to,” and “I annoy other people without meaning to.”

ED is common, but not unique to ADHD, and can also be found in other psychiatric disorders. However, there are a number of studies that support the concept of ED as a defining feature of ADHD (34–38). One of these studies showed that ED scores were significantly higher in adults with persistent ADHD than in those with remitted ADHD, suggesting a degree of developmental coherence (35). ED in combination with ADHD is thought to be associated with greater impairment in interpersonal relationships and occupational performance as well as with perceived lower quality of life compared to those with ADHD alone. (35, 39, 40). This finding remained significant after controlling for comorbid disorders (41). Previous studies have provided suggestive evidence to support the view that there is some genetic influence on the relationship between ADHD and ED (42, 43). However, this finding has not been replicated in other studies (44, 45): ADHD can be transmitted within families due to environmental factors; the presence of ADHD and ED does not increase the risk of ADHD for other siblings in the family. The genetic hypothesis now is that it is highly heritable. Taken together, although ED is not part of the diagnostic criteria, there is increasing evidence that ED should be considered as an important component that supports the diagnosis (46, 47).

With this in mind, we re-analyze the data from the above study to examine whether ED may affect the predictive value of predicting psychostimulant response in aADHD. First, based on the previous findings, we hypothesize that a significant proportion of participants in our data will have ED. Second, these participants may report more severe symptoms or differ in other clinical characteristics or in the regulation of brain arousal based on resting EEG. Finally, we investigated whether they responded differently to the medication.

## 2 Materials and methods

### 2.1 Participants

Data for the current study were collected from a previously published multicenter, single-arm, open-label clinical trial of participants with aADHD in Germany (25). This study was reviewed and approved by the local ethics committee (registration: EudraCT 2015–000,488–15; German Clinical Trial Register DRKS00009971, University of Leipzig Ethics Committee 337/15-ff). The methods utilized for participant inclusion and exclusion are described in detail elsewhere (25).

For analyses in the current study, participants’ data were included if they met the following criteria: ADHD diagnosis was confirmed by a psychiatrist and a psychologist according to clinical DSM-IV criteria during the screening period, no evidence of current suicidality, no acute anxiety or adjustment disorders, no history of substance abuse or dependence in the last 6 months, and no psychotic disorders. During the titration phase, all participants received extended-release methylphenidate for 4 weeks. Titration was started at 20 mg/day and then increased in 20 mg increments at weekly intervals to a weight-based target dose: i.e., 40 mg/day for participants weighing less than 55 kg, 60 mg/day for those weighing between 55 and 70 kg, and 80 mg/day for those weighing more than 70 kg. Exclusion criteria were evidence of an acute severe episode of major depression according to ICD-10 (i.e., ICD-10 Code F32.2, F32.3, F33.2, and F33.3), pathological activity or excessive artifacts in the EEG on the day before the titration (i.e., baseline) and after the titration (i.e., final visit). As a result, a total of 115 participants (mean age = 33.65, SD = 9.28; 37 women, 32.2%) were included in further analyses.

### 2.2 Study design and measurements

*ADHD-related symptoms* were measured by a set of self-report questionnaires: the short German Wender Utah Rating Scale [WURS-K; (48)] is a retrospective self-evaluation of ADHD-related symptoms at the age of approximately 8–10 years, a total score of 30 or above indicates probably existence of profound ADHD symptoms in childhood. The German version of Conners’ Adult ADHD Rating Scale (26) is a comprehensive assessment consisting of three symptom subscales and four factor-derived subscales. The three symptom subscales correspond to the respective DSM-IV criteria of the predominantly inattentive type (DSM-IA), the predominantly hyperactive–impulsive type (DSM-HYI), and the mixed type (DSM-G). A reduction of at least 30% reduction in the T-score of the latter subscale (DSM-G) was defined as successful treatment. The four factor-derived subscales are inattention/memory problems (IA/ME), emotional lability (IMP/EL), hyperactivity/restlessness (HY/RE), and self-concept problems (SC). The ADHD Index is a subscale of the CAARS that serves as a severity-based index to differentiate adults with ADHD from their non-clinical counterparts. The German Adult ADHD Self-Report Scale Symptom Checklist [ASRS v1.1; (49)] and the ADHD Self-Rating Questionnaire [ADHS-SB; (48)] are both consistent with DSM-IV criteria and address the manifestation of ADHD symptoms in adults.

*Emotional dysregulation (ED)* in this study was assessed by the sex- and age-corrected T-score of the emotional lability subscale (IMP/EL) of the CAARS. This 12-item subscale, which is administered in an identical format to the other CAARS scales mentioned above, assesses hot temper, tantrums, irritability, stress intolerance, and mood instability. Participants with high scores on this subscale are more likely to engage in impulsive acts, rapid mood changes, and irritability. According to the CAARS manual, T-scores above 60 could be a cause for concern and those above 70 indicate clinically relevant symptomatology. Therefore, participants with T-scores above 70 were considered clinically significantly atypical of the norm (ED+), whereas those with T-scores below 70 were considered normal or slightly atypical of the norm (ED-). Although the previous psychometric study of the CAARS supports the loading of impulsivity and emotional lability onto a common factor (50), a potential confound in this study was that this subscale contains items measuring impulsivity, and the relationship between ADHD and ED would subsequently be artificially inflated. Therefore, we conducted item correlations to assess the potential item overlap between the following scale/subscale pairs: CAARS IMP/EL subscale and BDI-II, CAARS IMP/EL subscale and DSM-IA, and CAARS IMP/EL subscale and DSM-HYI. A Spearman correlation coefficient greater than 0.4 indicates a strong relationship. The results are summarized in Supplementary Tables S1–S3. In the case of significant strong correlations, the result regarding the relationship between ADHD and ED should be interpreted with caution.

*Depressive symptoms*: clinical studies have documented that depression is one of the most common comorbidities in adults with ADHD (51, 52). Although we had already excluded participants with an acute severe episode of major depression from the study, there were a number of participants, who had mild or moderate episodes of major depression and/or subjectively reported depressive symptoms. The Beck Depression Inventory-II [BDI-II; (53)] measures these symptoms. At baseline, 39 participants had a BDI-II total score above 13, which is considered to be the cutoff for mild depression. An exploratory subgroup was created based on this result, considering that there are some items in the BDI-II (e.g., agitation and difficulty concentration) that could be understood as ADHD symptoms. At the suggestion of the reviewers, we created a frequency table to describe how participants answered each item on the BDI-II. The results are summarized in a Supplementary material.

In addition, a set of assessments was used to record other clinical-related impressions as well as current status. The Clinical Global Impression Severity Scale [CGI-S; (54)] was executed to externally rate the overall clinical severity of the participants’ illness at the time of assessment. The German Inventory of Interpersonal Problems [IIP; (55)] was used to identify participants’ most salient interpersonal difficulties. Subjectively perceived quality of life in various domains was measured using the short German version of the World Health Organization Quality of Life Questionnaire [WHOQOL-BREF; (56)].

### 2.3 Neurophysiological measurements

We recorded the EEG signal at baseline and the final visit with Ag/AgCl electrodes using a QuickAmp amplifier (Brain Products GmbH, Gilching, Germany) from 31 positions according to the 10–20 system in a 15-min resting condition. Thereafter, the EEG data were offline processed using a 0.5-70 Hz bandpass and a 50 Hz notch filter and then segmented into continuous 1-s segments. Brain arousal regulation (24) was assessed using VIGALL 2.1. One out of seven EEG-vigilance stages (stage 0, A1, A2, A3, B1, B2/3, and C) was automatically attributed to each artifact-free segment. Until now, the proportion of respective EEG-vigilance stages over the entire recording period was determined by the formula, i.e., amount * 100 / total number of artifact-free segments. The assigned EEG-vigilance stages were transformed into numerical values ranging from 7 (indexing stage 0, i.e., focused wakefulness) to 1 (indexing stage C, i.e., commencing sleep). For each 1-min EEG segment (60 1-s segments), a mean EEG-vigilance level was calculated by averaging assigned values of all artifact-free segments. There were theoretically 15 values for each participant to represent their changes in the EEG-vigilance level over the entire recording. The brain arousal regulation (i.e., speed and extent of the arousal decline over 15 min) was determined by the arousal stability score. The scoring criteria are described in detail and presented elsewhere (24). The arousal stability sores are reversely ranged from 11 (indicating rigid regulation of arousal) to 1 (indicating unstable regulation of arousal).

### 2.4 Statistical analysis

All analyses were conducted using IBM SPSS Statistics (Version 29.0, IBM Corp., Armonk, NY, United States) software. Measures of differences between the ED- and ED+ groups were conducted using the independent sample *t*-test (*T*) for metric and person chi-square (*Χ*^2^) test for two categorical nominal variables. Fisher’s exact test was used in the case of multidimensional categorical nominal variables. According to the results of the independent samples *t*-test for sex (see Supplementary Table S4), male and female participants showed significant differences in height, weight, scores on the CAARS subscales IMP/EL, HY/RE, DSM-HYI, DSM-G, ADHD-Index, ADHS-SB subscale impulsivity, and IIP. For these comparisons, we therefore used a one-way analysis of covariance (ANCOVA) to control for the effect of sex. To avoid overestimating the effect of ED due to possible regression to the mean when examining differences in response to treatment, we performed ANCOVA as suggested by Barnett et al. (57), with baseline scores as a covariate. For exploratory analyses, we performed a one-way analysis of variance (ANOVA) to compare sample means between ED+ with and without additional depressive symptoms. The t-tests or chi-squared statistics and number of degrees of freedom (df) were provided. The two-tailed significance level was set at a value of p of 0.05. The corrected significance level for multiple *post-hoc* tests according to Bonferroni was set at a value of p of 0.017. Cohen’s d or Hedge’s g (in case of unequal group variance using the pooled standard variance) and eta-squared (*η*^2^) were provided to evaluate the effect sizes of comparisons between groups. Furthermore, the number of sample sizes available for each test varied because some scale scores were not available due to missing data. As a result, we have always presented the number of sample sizes and df for each test in each table.

## 3 Results

### 3.1 How many participants reported symptoms of ED?

Table 1 shows the number of participants included in the final analyses (*n* = 115) at baseline. According to the T-score of the subscale IMP/EL of the CAARS, there were 56 (48.7%) participants in the ED+ group and the remaining 59 (51.3%) participants in the ED- group. Participants in the ED+ group were significantly older than those in the ED- group. No other demographic or clinical characteristics were significantly different.

**Table 1:** Comparisons of baseline demographic and clinical characteristics between ED+ and ED- groups.

	ED- (*n* = 59)	ED+ (*n* = 56)	All (*n* = 115)	Statistics, df	value of *p*	Effect size
Female, %	15, 25.4%	22, 39.3%	37, 32.2%	*Χ*^2^ = 2.53, 1	0.112	
Age (years)	31.4 ± 9.0	36.0 ± 9.1	33.6 ± 9.3	*T* = -2.69, 113	**0.008**	*d* = 0.50
Weight (kg)*	80.1 ± 17.0	79.5 ± 18.5	79.8 ± 17.6	*F* = 0.43, 1	0.513	*η*^2^ = 0.004
Height (cm)*	178.8 ± 8.8	175.0 ± 11.6	176.9 ± 10.4	*F* = 1.40, 1	0.239	*η*^2^ = 0.01
BMI (kg/m^2^)	25.0 ± 4.7	25.8 ± 4.7	25.4 ± 4.7	*T* = -0.91, 113	0.365	*d* = 0.17
Dosage (mg)				*Χ*^2^ = 1.67, 2	0.432	
40 mg, %	1.7%	5.4%	3.5%			
60 mg, %	25.4	30.4%	27.8%			
80 mg, %	72.9%	64.3%	68.7%			
Handedness				Fisher’s test	0.438	
Right-handed (%)	88.1%	92.9%	90.4%			
Left-handed (%)	8.5%	3.6%	6.3%			
Ambidextrous (%)	0%	1.8%	0.9%			
WURS-K	40.5 ± 11.3	47.4 ± 10.4	43.9 ± 11.4	*T* = -3.35, 110	**0.001**	*d* = 0.63
CAARS (*T*-score)						
IA/ME	75.9 ± 10.4	83.7 ± 7.8	79.7 ± 10.0	*T* = -4.54, 107.7	**<0.001**	*g* = 0.84
IMP/EL*	57.5 ± 8.3	80.2 ± 6.9	68.5 ± 13.7	*F* = 246.47, 1	**<0.001**	*η*^2^ = 0.69
HY/RE*	68.5 ± 13.6	75.3 ± 10.9	71.8 ± 12.7	*F* = 7.28, 1	**0.008**	*η*^2^ = 0.06
SC	63.3 ± 14.0	71.3 ± 11.6	67.1 ± 13.5	*T* = -3.31, 113	**0.001**	*d* = 0.62
DSM-IA	79.1 ± 9.9	84.6 ± 7.3	81.8 ± 9.1	*T* = -3.46, 107.0	**<0.001**	*g* = 0.64
DSM-HYI*	65.9 ± 10.7	78.4 ± 10.0	72.0 ± 12.1	*F* = 38.02, 1	**<0.001**	*η*^2^ = 0.25
DSM-G*	75.3 ± 9.4	84.8 ± 6.0	80.0 ± 9.2	*F* = 37.66, 1	**<0.001**	*η*^2^ = 0.25
ADHD-Index	72.5 ± 8.2	82.9 ± 5.9	77.6 ± 8.8	*T* = -7.81, 105.8	**<0.001**	*g* = 1.44
ADHD-SB (Sum)						
G	40.1 ± 7.3	45.1 ± 10.2	42.6 ± 9.2	*T* = -3.00, 113	**0.003**	*d* = 0.56
IA	17.2 ± 3.5	18.5 ± 4.8	17.8 ± 4.2	*T* = -1.67, 113	0.097	*d* = 0.31
HY	7.6 ± 3.3	8.8 ± 3.4	8.2 ± 3.4	*T* = -1.89, 113	0.061	*d* = 0.35
I*	6.1 ± 2.5	7.7 ± 2.6	6.9.2 ± 2.7	*F* = 9.89, 1	**0.002**	*η*^2^ = 0.08
ASRS (Sum)						
Part A	4.8 ± 0.9	5.0 ± 0.8	4.9 ± 0–9	*T* = -1.73, 111	0.086	*d* = 0.33
Part B	8.3 ± 2.2	10.0 ± 2.0	9.1 ± 2.3	*T* = -4.16, 113	**<0.001**	*d* = 0.78
BDI (Sum)	8.8 ± 7.4	14.6 ± 12.8	11.6 ± 10.7	*T* = -2.92, 87.3	**0.005**	*g* = 0.55
Subgroup (%)				*Χ*^2^ = 9.96, 1	**0.002**	
BDI > 13	12, 20.3% (range of score 15–35)	27, 48.2% (range of score 14–43)	39, 33.9% (range of score 14–43)			
BDI ≤ 13	47, 79.7% (range of score 0–13)	29, 51.8% (range of score 0–13)	76, 66.1% (range of score 0–13)			
CGI-S (Sum)	4.5 ± 1.1	4.8 ± 0.7	4.6 ± 0.9	*T* = -1.64, 90.1	0.105	*g* = 0.31
IIP (Sum)*	87.8 ± 32.1	116.0 ± 29.6	101.6 ± 33.9	*F* = 21.60, 1	**<0.001**	*η*^2^ = 0.16
WHOQOL-BREF (Sum)	256.1 ± 40.4	225.9 ± 50.6	241.4 ± 47.9	*T* = 3.55, 113	**<0.001**	*d* = 0.66
Physical health	66.0 ± 12.1	61.5 ± 15.8	63.8 ± 14.2	*T* = 1.69, 113	0.094	*d* = 0.32
Psychological health	57.1 ± 14.2	48.6 ± 17.4	53.0 ± 16.3	*T* = 2.90, 113	**0.005**	*d* = 0.54
Social relationship	60.2 ± 18.114.6	50.6 ± 19.7	55.5 ± 19.4	*T* = 2.72, 113	**0.008**	*d* = 0.51
Environment	72.8 ± 13.2	65.2±	69.1 ± 14.3	*T* = 2.95, 113	**0.004**	*d* = 0.55

Entries are mean ± standard deviation or numbers (%).

Bold fonts indicate statistical significance; the statistical significance value for these tests was set at 0.05.

*d* indicates effect sizes were estimated by Cohen’s *d*.

*g* indicates effect sizes were estimated by Hedge’s *g* in case of unequal group variance using the pooled standard variance.

*for these variables ANCOVA was executed with sex as co-variant.

### 3.2 Did the ED+ group have more severe symptoms than the ED- group?

As summarized in Table 1, the ED+ group reported significantly higher scores at baseline in ADHD-related ratings, higher depression scores, poorer quality of life, and more problems in interpersonal domains than the ED- group. Given the significant sex difference in some clinical characteristics (see Supplementary Table S4), an ANCOVA was performed. These results remained significant after controlling for sex. Considering the strong correlations between IMP/EL items and DSM-IA items (rho coefficients have a range from −0.090 to 0.437) and DSM-HYI (rho coefficients have a range from 0.001 to 0.614) subscales (see Supplementary Tables S1–S3), the results regarding the difference in core ADHD symptoms, especially hyperactivity/impulsivity symptoms, between the ED+ and ED- groups, should be interpreted with caution.

BDI-II was administered to assess the severity of comorbid depressive symptoms. In total, 48.2% of participants in the ED+ group reported a BDI-II total score above 13, which could be considered as having mild depression. In the ED- group, the proportion was 20.3%, resulting in a difference of 27.9 percentage points (*Χ*^2^ = 9.96, df = 1, *p* = 0.002). As summarized in Table 1, participants in the ED+ group reported subjectively more severe depressive symptoms than those in the ED- group (*T* = -2.92, df = 87.3, *p* = 0.005). We excluded participants with an acute severe episode of major depression according to ICD-10, and some items in the BDI-II (e.g., agitation and difficulty concentration) could be understood as ADHD symptoms. We created tables to describe the participants’ response behavior for each item on the BDI-II. These results are summarized in a Supplementary material. Descriptively, participants in the ED+ group experienced subjectively more severe specific depressive symptoms (e.g., loss of pleasure and loss of interest) that could not be understood as ADHD symptoms.

An exploratory analysis was conducted to examine whether the presence of comorbid depressive symptoms had an additional worsening effect on ADHD-related symptoms or other clinical characteristics. There was no further worsening effect on the ADHD symptoms as assessed by CAARS (0.300 ≤ *p* < 1.000). However, the ED+ with comorbid depressive symptoms showed lower quality of life in all domains (*p* ≤ 0.001) except social relationships (*p* = 0.135) and more interpersonal difficulties (*p* = 0.008). For more details, see Supplementary Table S5.

### 3.3 Were there peculiarities in the regulation of brain arousal based on resting EEG?

Table 2 shows the number of participants (*n* = 110) included in the final analyses in terms of EEG characteristics. Five participants in the ED+ group were excluded from this analysis due to excessive artifacts. No EEG characteristics differed significantly between the ED+ and ED- groups.

**Table 2:** Comparisons of baseline EEG characteristics between ED+ and ED- groups.

	ED- (*n* = 59)	ED+ (*n* = 51)	All (*n* = 110)	*T*-tests, degrees of freedom	Value of *p*	Effect size
EEG-vigilance stages (%)						
Stage 0	14.7 ± 16.2	17.7 ± 22.0	16.1 ± 19.1	−0.80, 108	0.424	d = 0.15
Stage A	54.2 ± 30.0	47.4 ± 31.9	51.0 ± 30.9	1.17, 108	0.245	d = 0.23
Stage A1	45.4 ± 31.3	41.5 ± 31.3	43.6 ± 31.3	0.66, 108	0.510	d = 0.13
Stage A2	5.4 ± 9.0	4.7 ± 6.8	5.1 ± 8.0	0.44, 108	0.658	d = 0.09
Stage A3	3.4 ± 8.7	1.2 ± 1.9	2.4 ± 6.6	1.93, 64.6	0.058	g = 0.34
Stage B1	14.4 ± 17.3	16.5 ± 21.5	15.4 ± 19.3	−0.58, 108	0.566	d = 0.11
Stage B2/3	8.9 ± 15.8	12.7 ± 14.7	10.7 ± 15.3	−1.29, 108	0.199	d = 0.25
Stage C	7.7 ± 14.4	5.8 ± 9.7	6.8 ± 12.4	0.82, 108	0.414	d = 0.16
Mean EEG-vigilance level	4.9 ± 1.2	4.8 ± 1.3	4.8 ± 1.3	0.15, 108	0.882	d = 0.03
Arousal stability score	6.1 ± 3.8	6.2 ± 3.8	6.1 ± 3.8	0.15, 108	0.918	d = 0.02

Entries are mean ± standard deviation or numbers (%).

Bold fonts indicate statistical significance; the statistical significance value for these tests was set at 0.05.

d indicates effect sizes were estimated by Cohen’s *d*.

*g* indicates effect sizes were estimated by Hedge’s g in case of unequal group variance using the pooled standard variance.

As the exploratory analysis shows, no EEG characteristics differed significantly among ED+ with and without comorbid depressive symptoms (0.040 ≤ *p* < 1.000, the corrected significance level for multiple *post-hoc* tests according to Bonferroni was set at a value of *p* of 0.017). Further details can be found in Supplementary Table S6.

### 3.4 Did they respond differently to the medication?

In the previous study (25), the therapy success was defined as a reduction of at least 30% in T-scores on the subscale DSM-G of the CAARS at the final visit. Using this definition, there were 16 (27.1%) and 18 (32.1%) responders in the ED- and ED+ groups, respectively, i.e., an absolute difference of −5 percentage points (*Χ*^2^ = 0.35, df = 1, *p* = 0.555). Additionally, we compared the percentage change in measurements between the ED+ and ED- groups. There were no significant differences in change (see Table 3) between the ED+ and ED- groups after controlling for the baseline scores.

**Table 3:** Differences in response to medication between ED+ and ED- groups.

Reduction in %	ED- (*n* = 48)	ED+ (*n* = 49)	All (*n* = 97)	ANCOVA, *F* (df = 1)	Value of *p*	Effect size *η*^2^
CAARS (*T*-score)						
IA/ME	−18.5 ± 15.0	−22.8 ± 20.0	−20.7 ± 17.7	0.38	0.541	0.004
IMP/EL	−14.7 ± 16.4	−26.4 ± 19.0	−20.6 ± 18.6	1.02	0.314	0.011
HY/RE	−21.8 ± 15.7	−25.1 ± 15.7	−23.5 ± 15.7	0.06	0.811	0.001
SC	−11.3 ± 19.4	−17.2 ± 21.6	−14.3 ± 20.7	0.04	0.846	4.0E-4
DSM-IA	−21.9 ± 18.0	−23.1 ± 17.2	−22.5 ± 17.5	0.08	0.785	0.001
DSM-HYI	−16.8 ± 15.1	−24.2 ± 16.6	−20.5 ± 16.2	0.002	0.961	2.5E-5
DSM-G	−22.2 ± 15.9	−25.4 ± 16.4	−23.9 ± 16.2	0.22	0.638	0.002
ADHD-index	−19.9 ± 16.1	−26.1 ± 18.2	−23.0 ± 17.4	0.56	0.454	0.006
BDI (Sum)	−4.6 ± 112.3	−41.8 ± 43.8	−23.6 ± 86.1	1.56	0.215	0.018
CGI-S (Sum)	−18.6 ± 37.5	−27.7 ± 16.8	−23.3 ± 29.1	0.46	0.501	0.005
IIP (Sum)	−3.7 ± 51.9	−16.4 ± 41.5	−9.8 ± 47.4	0.14	0.710	0.002
WHOQOL-BREF (Sum)	5.9 ± 18.3	15.7 ± 31.1	10.9 ± 25.9	0.51	0.476	0.005
Physical health	6.9 ± 20.7	18.9 ± 47.0		0.38	0.539	0.004
Psychological health	16.3 ± 32.6	32.3 ± 60.3		0.04	0.845	4.0E-4
Social relationship	5.0 ± 36.0	31.3 ± 129.3		0.29	0.606	0.003
Environment	2.5 ± 15.0	2.2 ± 30.3		1.02	0.315	0.010

ANCOVA was executed with measurement score at baseline as co-variant.

Entries are mean ± standard deviation.

**Figure 1:**
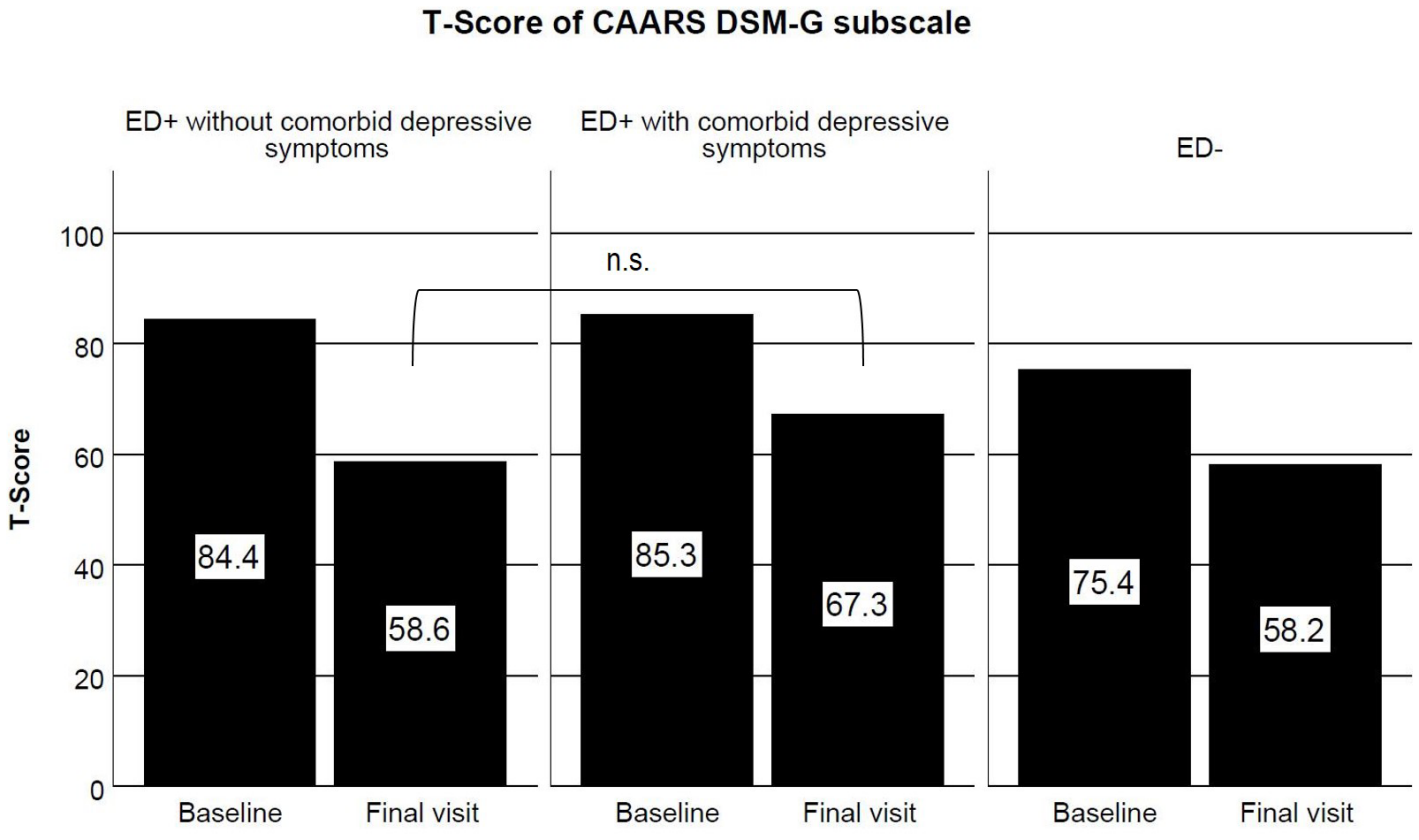
*T*-scores of DSM-G subscale of CAARS at baseline and final visit in ED+ without comorbid depressive symptoms (left panel), ED+ with comorbid depressive symptoms (middle panel) and ED- (right panel) groups.

According to the explorative analysis, within the ED+ group, there were 12 participants (41.4%) without comorbid depressive symptoms classified as responders, while the amount for those with comorbid depressive symptoms was 6 (22.2%), i.e., an absolute difference of 19.2 percentage points (*Χ*^2^ = 2.35, df = 1, *p* = 0.125). As shown in Figure 1, ED+ with and without comorbid depressive symptoms showed similar severity of ADHD symptoms at baseline (estimated mean difference = 1.5, 95% CI -3.7–6.7, *p* = 1.000). Descriptively, the former had 8.7 points more than the latter in *t*-scores of the DSM-G subscale of the CAARS after treatment (*F* = 4.89, df = 1, *p* = 0.032, not significant as compared to the corrected value of *p* of 0.017 due to multiple comparisons).

**Figure 2:**
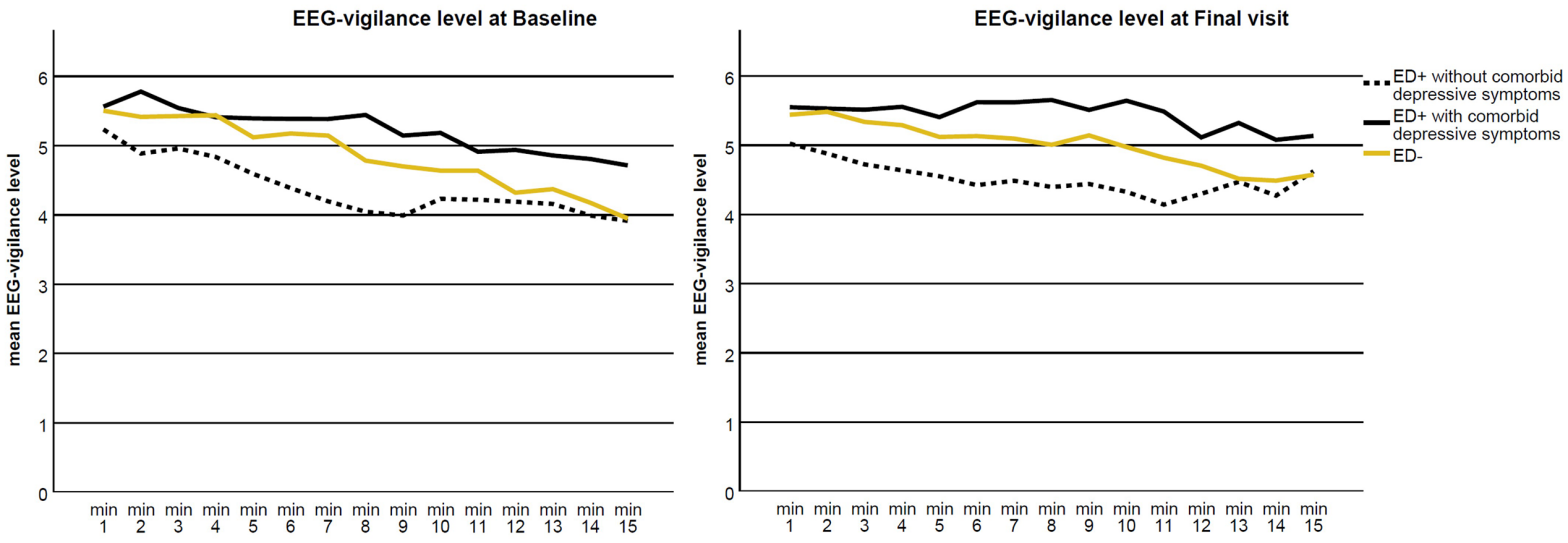
Mean EEG-vigilance level in 1-min blocks at baseline (before the therapy; left) and final visit (after the therapy; right) for the ED+ without comorbid depressive symptoms (dark line), ED+ with comorbid depressive symptoms (dotted line) and ED- (light line) groups.

Figure 2 illustrates the EEG-vigilance level at baseline (before the treatment) and at the final visit (after the treatment). At baseline, all groups showed a trend of a slight decline in brain arousal regulation over the recording period, with ED+ with comorbid depressive symptoms being somewhat more vigilant in terms of higher EEG-vigilance level compared to ED+ without comorbid depressive symptoms. The slope of the EEG-vigilance level of participants in the ED+ group with comorbid depressive symptoms became somewhat flatter after treatment, indicating a trend toward stable brain arousal regulation due to the therapy. However, there was no significant difference in the change in EEG-vigilance level between these two subgroups (*F* = 1.41, df = 1, *p* = 0.242, not significant as compared to the corrected value of *p* of 0.017 due to multiple comparisons).

## 4 Discussion

The aim of the current study was to examine whether the symptoms of emotional dysregulation (ED) in an aADHD sample affect the severity of ADHD symptoms based on self-report, brain arousal regulation via resting EEG, and response to the medication. ED was assessed by the emotional lability subscale (IMP/EL) of the CAARS.

We found that approximately 48% of aADHD in our sample were rated as having ED. Previous studies have estimated that approximately 34–70% of aADHD may have difficulties in their emotional regulation [ER; (58–60)]. Reimherr et al. (61) even identified 72% of subjects with ED in a population of aADHD. The relatively lower prevalence in our sample may be due to our ED assessment tool. There was a considerable number of studies (36, 62) that assessed ED via temper control, affective lability, and emotional overreactivity subscales of the Wender Reimherr adult attention-deficit disorder scale (48). Nevertheless, there are also studies using the same measurement as this study. However, there are no studies that separate participants into a subgroup whose ED is clinically significantly atypical of the norm, based on the *T*-score in this subscale. In addition, we have included only those participants with a *T*-score above 70 (indicating clinically relevant symptomatology in this subscale) in the ED+ group, and those with a *T*-score above 60 but below 70 (a range that might be of concern) in the ED- group, which might result in a relatively lower prevalence compared to what is usually found. Furthermore, the range in the reported prevalence of ED in aADHD is wide. This is most likely due to the lack of a consensual definition of ED and the refined definition of instruments used to measure ED. With respect to the definition of ED, it is generally considered a dimensional construct containing intrinsic and extrinsic adaptive processes. A meta-analysis study (27) identified three main dimensions of ED based on the narrative synthesis: emotional recognition, emotional lability, and negative emotional response. In another empirical article (39), the authors derived four components, namely being aware of emotions, making sense of emotions, modifying and accepting emotions, and confronting emotions with self-encouragement by principle components analysis assembling ER. Nevertheless, regardless of the non-consensual refined definition and operationalization, given the fact that ED is frequently reported in numerous studies, it could be an important feature of ADHD.

We showed (Table 1) that aADHD with ED was more impaired in terms of severity of inattentive symptoms, comorbid depressive symptoms, interpersonal relationships, and quality of life. This finding is partially supported by a recent study (62). The authors found that the presence of ED proved to be an indicator of the severity of aADHD, independent of the presence of comorbidity. In addition, our participants reported subjectively more impaired hyperactive/impulsive symptoms. As there were strong correlations between 7 out of 12 items in the IMP/EL subscale and the items in the DSM-HYI subscale (see Supplementary Table S2), the results regarding participants in the ED+ group showing more severe hyperactive/impulsive symptoms should be interpreted with caution. The IMP/EL subscale measures not only ED-like symptoms but also symptoms of impulsivity (e.g., “I blurt out things,” and “I say things without thinking”). Therefore, the difference in severity of hyperactive/impulsive symptoms could be increased by overlapping items between the IMP/El and the DSM-HYI subscales. In our study, ED may be simultaneously or similarly associated with the severity of inattentive symptoms and the development of later depression.

In addition, aADHD participants with ED were more likely to score at the cutoff point (which could be considered mild depression) on the BDI-II and reported descriptively more severe specific depressive symptoms (e.g., loss of interest and loss of pleasure) that could not be considered as ADHD symptoms. ADHS is a neurodevelopmental disorder, 40–77% of children with ADHD continue to show symptoms into adulthood (2–6). Mood disorders such as depression are one of the most common comorbidities in aADHD (52, 63, 64). Deficient ER in youth with ADHD was demonstrated as a possible common risk factor contributing to subsequent depression (67). Additionally, aADHD with ED reported more severe ADHD symptoms in childhood based on a retrospective assessment (i.e., WURS-K). If ED is seen already in childhood, depressive symptoms could be induced due to poor ER strategies (e.g., self-blame and catastrophizing or rumination), which in turn cause exacerbation of ADHD symptoms and more functioning impairments. This could consequently contribute to the persistence of ADHD and the manifestation of depression with increasing age.

Our results (Table 2) provided no evidence that ED affects brain arousal regulation. To the best of our knowledge, this is the first study examining brain arousal regulation regarding ED in aADHD. To date, it is unclear whether the EEG can discriminate between aADHD with and without ED. A recent systematic review (15) questioned the use of EEG in aADHD diagnosis due to inconsistent findings, a range of other psychiatric diagnoses, i.e., comorbid disorders might account for the inconsistent findings. In the absence of a control group, our results cannot be used as conclusive evidence of the sensitivity of EEG as a diagnostic tool in general. However, our findings provide evidence that EEG may not be a useful diagnostic tool for specifying subtypes of aADHD, particularly in the presence of comorbid depressive symptoms.

A previous study compared ED and autonomic nervous system (ANS) function in the parasympathetic (indexed by respiratory sinus arrhythmia, RSA) and sympathetic systems between children with and without ADHD (66), during induction and suppression of negative and positive emotions. Children without ADHD showed systemic variation in RSA according to emotional valence, while ADHD children displayed a stable pattern of elevated RSA across all conditions. This pattern was not attributed to pre-existing ANS functioning in resting and mood conditions prior to the task. In other words, ADHD children showed trait-like similar physiological functioning to typically developing children but a stable pattern in terms of an elevated physiological response to transient emotional stimuli. Morris et al. (67) replicated this finding. The ANS has been shown to vary with the state of brain arousal, and one of the previous studies (21) found a consistent decrease in ANS activity with decreasing brain arousal. Based on these findings, we assumed aADHD with and without ED displayed similar brain arousal regulation at rest in the absence of external or internal interfering stimuli. However, for aADHD with ED, it could be transiently either more stable or unstable at a certain moment when they are distracted by their own attention to a negative or positive memory. The arousal stability score is an overall assessment of the speed/extent of arousal decline over 15 min and is therefore not able to capture this kind of transient change. However, if aADHD with ED reported already depressive symptoms prior to the test condition, then it is possible that the brain arousal regulation during the recording period was masked by a state-like depression and thus became more stable.

There was no evidence that ED affected response to the medication in this study (*p* = 0.555). The percentage reductions in ADHD symptoms, depression-related symptoms, impairments in interpersonal relationships, and quality of life were similar between the two groups (Table 3). This result suggests that there may be no difference in response to therapy that could be attributed to the effects of ED. These findings are consistent with previous results. In the study of Reimherr et al. (61), they demonstrated that their ED + ADHD patients responded at least as well as the non-ED patients. For the exploratory aim, we compared further differences in change in ADHD symptom severity based on the CAARS DSM-G between ED with and without mixed comorbid depressive symptoms (Figure 1). At a descriptive level, there was a difference between these two subgroups even after controlling for baseline scores, suggesting a potential negative impact of ED with comorbid depressive symptoms on the treatment response. However, this was not retained after value of p correction due to multiple comparisons.

As mentioned above, EEG-based brain arousal regulation may not be a useful diagnostic tool for specifying subtypes of aADHD in the presence of comorbid depressive symptoms. However, there was a trend shown by the subgroup of this study, which displayed a change in brain arousal regulation in association with symptom improvement. Figure 2 shows brain arousal regulation in aADHD with ED and additional depressive symptoms flattening out after therapy, indicating that brain arousal regulation became more stable. In line with the brain arousal regulation model (18), ADHD has long been discussed as hypoaroused by several research groups (16, 68, 69). A rapid decline in arousal over a short period of time has been empirically demonstrated in ADHD (16, 19). Conversely, hyperstable arousal regulation has been proposed for patients with unipolar major depression (18) and demonstrated in the following studies (70–72). If reported depressive symptoms masked the brain arousal regulation in subgroups of aADHD with ED and made it more stable, a hyperstable regulation of brain arousal could be expected in cases of ADHD symptom improvement as the remaining depressive symptoms moved forward and the brain arousal regulation became hyperstable (Figure 2).

We acknowledge some limitations of this study. First, the participants were recruited from outpatients in a clinical setting; there may be individuals with severe functional impairments or requiring healthcare due to the relatively short duration of treatment and lack of blinded assessment of treatment outcomes; and the rate of defined treatment success was low. Second, as this study was a reanalysis of existing data, it was not possible to select instruments to measure emotional dysregulation or depressive symptoms, resulting in some item correlations between subscales, which in turn limits the generalizability of our results.

## 5 Conclusion

Taken together, this study provides evidence to consider ED as a significant feature of ADHD by demonstrating that a significant proportion in the current sample of aADHD have ED which consequently leads to more impairments in different domains. ED does not affect EEG-based brain arousal regulation and response to medication, and regardless of the presence of comorbid depressive symptoms, both groups showed a similar type of arousal regulation, ED+ responding as well as those ED-. aADHD with mixed ED and comorbid depression may affect treatment outcomes.

## Data availability statement

The original contributions presented in the study are included in the article/Supplementary materialS1, further inquiries can be directed to the corresponding author/s.

## Ethics statement

The studies involving humans were reviewed and approved by the local ethics committee (registration: EudraCT 2015–000,488–15; German Clinical Trial Register DRKS00009971, University of Leipzig Ethics Committee 337/15-ff). The studies were conducted in accordance with the local legislation and institutional requirements. The participants provided their written informed consent to participate in this study.

## Author contributions

JH: Conceptualization, Formal analysis, Methodology, Visualization, Writing – original draft, Writing – review & editing. MN: Conceptualization, Methodology, Validation, Writing – review & editing. EA: Writing – review & editing. HB: Writing – review & editing. PB: Writing – review & editing. TE: Writing – review & editing. AF: Writing – review & editing. JG: Writing – review & editing. IH: Writing – review & editing. KH: Writing – review & editing. SK-S: Writing – review & editing. AR: Writing – review & editing. DS: Writing – review & editing. SU: Writing – review & editing. MS: Conceptualization, Project administration, Supervision, Validation, Writing – review & editing.
