# Supplementary material for: The impact of emotional dysregulation and comorbid depressive symptoms on clinical features, brain arousal, and treatment response in adults with ADHD
Source: Front Psychiatry. 2024 Jan 5;14:1294314. doi: 10.3389/fpsyt.2023.1294314 (PMC10797130; doi:10.3389/fpsyt.2023.1294314)
Supplement: Supplementary file 1 [file Table_1.DOCX]

The impact of emotional dysregulation and comorbid depressive symptoms on clinical features. brain arousal and treatment response in adults with ADHD

# Jue Huang1, Mauche Nicole1, Eike Ahlers2, Holger Bogatsch3, Pierre Böhme4, Thomas Ethofer5,6, Andreas J Fallgatter6,7, Jürgen Gallinat8, Ulrich Hegerl9, Isabella Heuser2, Knut Hoffmann4, Sarah Kittel-Schneider9,10, Andreas Reif9, Daniel Schöttle8, Stefan Unterecker10, and Maria Strauß1*

1 Department of Psychiatry and Psychotherapy, University of Leipzig, Leipzig, 04103, Germany

2 Department of Psychiatry and Psychotherapy, Charité – Universitätsmedizin Berlin, Berlin, 10117, Germany

3 Clinical Trial Centre Leipzig, Faculty of Medicine, University of Leipzig, Leipzig, 04107, Germany

4 Department of Psychiatry Psychotherapy and Preventive Medicine, University Hospital of Bochum, Bochum, 44791, Germany

5 Department of Biomedical Magnetic Resonance, University Hospital of Tübingen, Tübingen, 72076, Germany

6 Department of Psychiatry and Psychotherapy, University Hospital of Tübingen, Tübingen, 72076, Germany

7 Tübingen Center for Mental Health (TüCMH), University Hospital of Tübingen, Tübingen, 72076, Germany

8 Department of Psychiatry and Psychotherapy, University Medical Center Hamburg-Eppendorf, Hamburg, 20251, Germany

9 Department of Psychiatry, Psychotherapy and Psychosomatic Medicine, University Hospital of Frankfurt – Goethe University, Frankfurt am Main, 60528, Germany

10 Department of Psychiatry, Psychosomatics and Psychotherapy, University Hospital of Würzburg, Würzburg, 97080, Germany

* Corresponding author:

Maria Strauß, MD, MHBA

University Hospital of Leipzig, Department of Psychiatry and Psychotherapy

Semmelweisstraße 10, Haus 13, 04103 Leipzig, Germany

Phone: +49 341 97 24334

Email: [Maria.Strauss@medizin.uni-leipzig.de](mailto:Maria.Strauss@medizin.uni-leipzig.de) (ORCID: 0000-0002-0072-2199)

Contributing authors:

[Jue.Huang@medizin.uni-leipzig.de](mailto:Jue.Huang@medizin.uni-leipzig.de) (ORCID: 0000-0003-1830-9340);

[Nicole.Mauche@medizin.uni-leipzig.de](mailto:Nicole.Mauche@medizin.uni-leipzig.de) (ORCID: 0000-0002-4223-5030);

[Eike.Ahlers@charite.de](mailto:Eike.Ahlers@charite.de);

[holger.bogatsch@zks.uni-leipzig.de](mailto:holger.bogatsch@zks.uni-leipzig.de);

[pierre.boehme@lwl.org](mailto:pierre.boehme@lwl.org);

[Thomas.Ethofer@med.uni-tuebingen.de](mailto:Thomas.Ethofer@med.uni-tuebingen.de);

[andreas.fallgatter@med.uni-tuebingen.de](mailto:andreas.fallgatter@med.uni-tuebingen.de);

[j.gallinat@uke.de](mailto:j.gallinat@uke.de);

[ulrich.hegerl@deutsche-depressionshilfe.de](mailto:ulrich.hegerl@deutsche-depressionshilfe.de);

[katharina.schmalfeld@charite.de](mailto:katharina.schmalfeld@charite.de);

[knut.hoffmann@lwl.org](mailto:knut.hoffmann@lwl.org);

[Kittel_S@ukw.de](mailto:Kittel_S@ukw.de);

[andreas.reif@kgu.de](mailto:andreas.reif@kgu.de) (ORCID: 0000-0002-0992-634X);

[d.schoettle@uke.de](mailto:d.schoettle@uke.de);

[unterecker_s@ukw.de](mailto:unterecker_s@ukw.de);

[Maria.Strauss@medizin.uni-leipzig.de](mailto:Maria.Strauss@medizin.uni-leipzig.de) (ORCID: 0000-0002-0072-2199)

**Table S1. Item correlations between the subscale IMP/EL and DSM-IA of the CAARS**

| Correlation |  | CAARS Item4 | CAARS Item8 | CAARS Item12 | CAARS Item19 | CAARS Item23 | CAARS Item30 | CAARS Item35 | CAARS Item39 | CAARS Item43 | CAARS Item47 | CAARS Item52 | CAARS Item61 |
| --- | --- | --- | --- | --- | --- | --- | --- | --- | --- | --- | --- | --- | --- |
| CAARS Item 2 | rho | 0.179 | 0.150 | 0.039 | 0.065 | 0.140 | .319** | 0.179 | 0.099 | 0.082 | 0.128 | .240* | 0.080 |
|  | Sig. (2-tailed) | 0.057 | 0.112 | 0.679 | 0.493 | 0.137 | 0.001 | 0.057 | 0.295 | 0.386 | 0.175 | 0.010 | 0.401 |
|  | N | 114 | 114 | 113 | 113 | 114 | 114 | 114 | 113 | 114 | 114 | 114 | 113 |
| CAARS Item 24 | rho | 0.047 | .243** | 0.061 | -0.042 | -0.017 | 0.138 | 0.056 | 0.013 | -0.090 | 0.006 | -0.072 | -0.061 |
|  | Sig. (2-tailed) | 0.619 | 0.009 | 0.517 | 0.655 | 0.853 | 0.143 | 0.549 | 0.891 | 0.338 | 0.947 | 0.445 | 0.519 |
|  | N | 115 | 115 | 114 | 114 | 115 | 115 | 115 | 114 | 115 | 115 | 115 | 114 |
| CAARS Item 29 | rho | 0.126 | .277** | 0.015 | 0.158 | .240** | .437** | 0.130 | .235* | 0.176 | 0.069 | .280** | .189* |
|  | Sig. (2-tailed) | 0.181 | 0.003 | 0.877 | 0.093 | 0.010 | 0.000 | 0.167 | 0.012 | 0.060 | 0.463 | 0.002 | 0.043 |
|  | N | 115 | 115 | 114 | 114 | 115 | 115 | 115 | 114 | 115 | 115 | 115 | 114 |
| CAARS Item 33 | rho | .264** | .303** | .193* | 0.054 | 0.120 | .203* | .247** | .278** | .256** | .191* | .235* | 0.183 |
|  | Sig. (2-tailed) | 0.004 | 0.001 | 0.040 | 0.569 | 0.200 | 0.029 | 0.008 | 0.003 | 0.006 | 0.040 | 0.011 | 0.052 |
|  | N | 115 | 115 | 114 | 114 | 115 | 115 | 115 | 114 | 115 | 115 | 115 | 114 |
| CAARS Item 42 | rho | 0.148 | 0.175 | .198* | 0.135 | 0.144 | .191* | .269** | .189* | .203* | 0.022 | 0.177 | 0.114 |
|  | Sig. (2-tailed) | 0.115 | 0.061 | 0.035 | 0.152 | 0.124 | 0.041 | 0.004 | 0.044 | 0.029 | 0.813 | 0.058 | 0.228 |
|  | N | 115 | 115 | 114 | 114 | 115 | 115 | 115 | 114 | 115 | 115 | 115 | 114 |
| CAARS Item 48 | rho | .222* | .412** | .333** | .221* | .261** | .195* | .308** | 0.168 | 0.170 | .366** | .218* | .248** |
|  | Sig. (2-tailed) | 0.018 | 0.000 | 0.000 | 0.019 | 0.005 | 0.038 | 0.001 | 0.075 | 0.071 | 0.000 | 0.020 | 0.008 |
|  | N | 114 | 114 | 113 | 113 | 114 | 114 | 114 | 113 | 114 | 114 | 114 | 113 |
| CAARS Item 60 | rho | 0.103 | .213* | -0.012 | 0.002 | 0.043 | 0.081 | -0.018 | 0.069 | 0.045 | 0.070 | 0.097 | 0.046 |
|  | Sig. (2-tailed) | 0.272 | 0.022 | 0.896 | 0.982 | 0.651 | 0.389 | 0.851 | 0.463 | 0.634 | 0.460 | 0.301 | 0.627 |
|  | N | 115 | 115 | 114 | 114 | 115 | 115 | 115 | 114 | 115 | 115 | 115 | 114 |
| CAARS Item 64 | rho | 0.102 | .235* | 0.091 | 0.061 | 0.095 | .415** | 0.162 | 0.033 | 0.062 | 0.135 | .193* | 0.128 |
|  | Sig. (2-tailed) | 0.276 | 0.012 | 0.333 | 0.518 | 0.312 | 0.000 | 0.084 | 0.730 | 0.507 | 0.150 | 0.039 | 0.175 |
|  | N | 115 | 115 | 114 | 114 | 115 | 115 | 115 | 114 | 115 | 115 | 115 | 114 |
| CAARS Item 65 | rho | .207* | .391** | 0.145 | .226* | .292** | .239** | .207* | 0.123 | 0.182 | 0.170 | .237* | .279** |
|  | Sig. (2-tailed) | 0.026 | 0.000 | 0.123 | 0.016 | 0.002 | 0.010 | 0.026 | 0.191 | 0.051 | 0.070 | 0.011 | 0.003 |
|  | N | 115 | 115 | 114 | 114 | 115 | 115 | 115 | 114 | 115 | 115 | 115 | 114 |
| ** p<0.01 two-tailed. | |  |  |  |  |  |  |  |  |  |  |  |  |
| * p<0.05 two-tailed. | |  |  |  |  |  |  |  |  |  |  |  |  |
| row: items of the IMP/EL subsclae | | |  |  |  |  |  |  |  |  |  |  |  |
| column: items of the DSM-IA subscale | | |  |  |  |  |  |  |  |  |  |  |  |

**Table S2. Item correlations between the subscale IMP/EL and DSM-HYI of the CAARS**

| Correlation |  | CAARS Item4 | CAARS Item8 | CAARS Item12 | CAARS Item19 | CAARS Item23 | CAARS Item30 | CAARS Item35 | CAARS Item39 | CAARS Item43 | CAARS Item47 | CAARS Item52 | CAARS Item61 |
| --- | --- | --- | --- | --- | --- | --- | --- | --- | --- | --- | --- | --- | --- |
| CAARS Item 9 | rho | .614** | .198* | .588** | .330** | 0.175 | 0.179 | .532** | .436** | .468** | .202* | .326** | .271** |
|  | Sig. (2-tailed) | 0.000 | 0.034 | 0.000 | 0.000 | 0.062 | 0.055 | 0.000 | 0.000 | 0.000 | 0.031 | 0.000 | 0.004 |
|  | N | 115 | 115 | 114 | 114 | 115 | 115 | 115 | 114 | 115 | 115 | 115 | 114 |
| CAARS Item 14 | rho | .502** | .200* | .543** | .324** | 0.112 | 0.129 | .292** | .353** | .430** | .358** | .420** | .252** |
|  | Sig. (2-tailed) | 0.000 | 0.032 | 0.000 | 0.000 | 0.235 | 0.169 | 0.002 | 0.000 | 0.000 | 0.000 | 0.000 | 0.007 |
|  | N | 115 | 115 | 114 | 114 | 115 | 115 | 115 | 114 | 115 | 115 | 115 | 114 |
| CAARS Item 21 | rho | .320** | 0.177 | .286** | 0.129 | 0.161 | .340** | .187* | .196* | .251** | .402** | .303** | .236* |
|  | Sig. (2-tailed) | 0.000 | 0.058 | 0.002 | 0.170 | 0.086 | 0.000 | 0.045 | 0.037 | 0.007 | 0.000 | 0.001 | 0.011 |
|  | N | 115 | 115 | 114 | 114 | 115 | 115 | 115 | 114 | 115 | 115 | 115 | 114 |
| CAARS Item 22 | rho | .356** | .312** | .320** | .330** | .326** | .218* | .292** | .198* | .300** | .371** | .253** | .348** |
|  | Sig. (2-tailed) | 0.000 | 0.001 | 0.001 | 0.000 | 0.000 | 0.019 | 0.002 | 0.034 | 0.001 | 0.000 | 0.006 | 0.000 |
|  | N | 115 | 115 | 114 | 114 | 115 | 115 | 115 | 114 | 115 | 115 | 115 | 114 |
| CAARS Item 38 | rho | .238* | 0.068 | .293** | 0.121 | 0.139 | 0.163 | 0.179 | .328** | .314** | 0.161 | .243* | .211* |
|  | Sig. (2-tailed) | 0.013 | 0.484 | 0.002 | 0.215 | 0.151 | 0.092 | 0.064 | 0.001 | 0.001 | 0.095 | 0.011 | 0.029 |
|  | N | 108 | 108 | 107 | 107 | 108 | 108 | 108 | 108 | 108 | 108 | 108 | 107 |
| CAARS Item 41 | rho | 0.145 | .224* | 0.155 | .258** | .232* | 0.094 | 0.090 | 0.081 | 0.093 | .204* | .214* | 0.176 |
|  | Sig. (2-tailed) | 0.124 | 0.017 | 0.100 | 0.006 | 0.013 | 0.320 | 0.343 | 0.392 | 0.323 | 0.029 | 0.022 | 0.062 |
|  | N | 114 | 114 | 113 | 113 | 114 | 114 | 114 | 113 | 114 | 114 | 114 | 113 |
| CAARS Item 50 | rho | 0.162 | 0.038 | 0.107 | 0.060 | -0.050 | 0.029 | 0.133 | 0.001 | -0.045 | 0.057 | 0.014 | 0.055 |
|  | Sig. (2-tailed) | 0.083 | 0.688 | 0.255 | 0.526 | 0.597 | 0.755 | 0.156 | 0.992 | 0.636 | 0.546 | 0.883 | 0.564 |
|  | N | 115 | 115 | 114 | 114 | 115 | 115 | 115 | 114 | 115 | 115 | 115 | 114 |
| CAARS Item 58 | rho | .594** | .229* | .474** | .229* | 0.159 | 0.150 | .584** | .393** | .345** | .295** | .375** | .295** |
|  | Sig. (2-tailed) | 0.000 | 0.015 | 0.000 | 0.015 | 0.093 | 0.113 | 0.000 | 0.000 | 0.000 | 0.002 | 0.000 | 0.001 |
|  | N | 113 | 113 | 112 | 112 | 113 | 113 | 113 | 112 | 113 | 113 | 113 | 113 |
| CAARS Item 62 | rho | .576** | .273** | .556** | .365** | .264** | .272** | .509** | .492** | .460** | .433** | .559** | .327** |
|  | Sig. (2-tailed) | 0.000 | 0.003 | 0.000 | 0.000 | 0.005 | 0.003 | 0.000 | 0.000 | 0.000 | 0.000 | 0.000 | 0.000 |
|  | N | 114 | 114 | 113 | 113 | 114 | 114 | 114 | 113 | 114 | 114 | 114 | 114 |
| ** p<0.01 two-tailed. | |  |  |  |  |  |  |  |  |  |  |  |  |
| * p<0.05 two-tailed. | |  |  |  |  |  |  |  |  |  |  |  |  |
| row: items of the IMP/EL subscale | | |  |  |  |  |  |  |  |  |  |  |  |
| column: items of the DSM-HYI subscale | | |  |  |  |  |  |  |  |  |  |  |  |

**Table S3. Item correlations between the subscale IMP/EL of the CAARS and items of the BDI-II**

| Correlation | | CAARS Item4 | CAARS Item8 | CAARS Item12 | CAARS Item19 | CAARS Item23 | CAARS Item30 | CAARS Item35 | CAARS Item39 | CAARS Item43 | CAARS Item47 | CAARS Item52 | CAARS Item61 |
| --- | --- | --- | --- | --- | --- | --- | --- | --- | --- | --- | --- | --- | --- |
| BDI Item 1 | rho | 0.072 | 0.181 | 0.055 | 0.005 | .198* | -0.065 | 0.121 | 0.137 | 0.030 | .226* | 0.085 | .220* |
|  | Sig. (2-tailed) | 0.446 | 0.052 | 0.563 | 0.956 | 0.034 | 0.493 | 0.198 | 0.147 | 0.752 | 0.015 | 0.368 | 0.019 |
|  | N | 115 | 115 | 114 | 114 | 115 | 115 | 115 | 114 | 115 | 115 | 115 | 114 |
| BDI Item 2 | rho | -0.082 | 0.071 | -0.046 | -0.069 | 0.114 | -0.065 | 0.038 | 0.028 | -0.079 | 0.177 | 0.125 | 0.130 |
|  | Sig. (2-tailed) | 0.384 | 0.448 | 0.625 | 0.466 | 0.223 | 0.491 | 0.685 | 0.770 | 0.401 | 0.058 | 0.185 | 0.169 |
|  | N | 115 | 115 | 114 | 114 | 115 | 115 | 115 | 114 | 115 | 115 | 115 | 114 |
| BDI Item 3 | rho | 0.142 | .266** | .222* | 0.003 | 0.127 | 0.049 | 0.152 | .222* | 0.125 | .258** | .236* | 0.104 |
|  | Sig. (2-tailed) | 0.132 | 0.004 | 0.018 | 0.973 | 0.177 | 0.608 | 0.106 | 0.018 | 0.185 | 0.006 | 0.012 | 0.272 |
|  | N | 114 | 114 | 113 | 113 | 114 | 114 | 114 | 113 | 114 | 114 | 114 | 113 |
| BDI Item 4 | rho | 0.057 | 0.106 | 0.166 | 0.119 | 0.160 | -0.084 | 0.084 | 0.178 | 0.111 | .275** | 0.097 | .262** |
|  | Sig. (2-tailed) | 0.545 | 0.261 | 0.078 | 0.207 | 0.087 | 0.370 | 0.374 | 0.058 | 0.237 | 0.003 | 0.302 | 0.005 |
|  | N | 115 | 115 | 114 | 114 | 115 | 115 | 115 | 114 | 115 | 115 | 115 | 114 |
| BDI Item 5 | rho | 0.129 | 0.131 | 0.143 | 0.045 | 0.084 | 0.040 | 0.148 | 0.130 | 0.115 | .216* | .243** | 0.064 |
|  | Sig. (2-tailed) | 0.170 | 0.163 | 0.130 | 0.632 | 0.371 | 0.675 | 0.114 | 0.169 | 0.220 | 0.021 | 0.009 | 0.499 |
|  | N | 115 | 115 | 114 | 114 | 115 | 115 | 115 | 114 | 115 | 115 | 115 | 114 |
| BDI Item 6 | rho | 0.131 | 0.156 | 0.091 | 0.126 | 0.142 | 0.167 | 0.157 | 0.147 | 0.078 | .232* | .263** | 0.183 |
|  | Sig. (2-tailed) | 0.162 | 0.097 | 0.337 | 0.180 | 0.129 | 0.075 | 0.093 | 0.119 | 0.405 | 0.013 | 0.005 | 0.051 |
|  | N | 115 | 115 | 114 | 114 | 115 | 115 | 115 | 114 | 115 | 115 | 115 | 114 |
| BDI Item 7 | rho | 0.041 | 0.182 | .188* | 0.176 | .287** | 0.130 | 0.139 | .204* | 0.164 | .246** | .338** | .203* |
|  | Sig. (2-tailed) | 0.666 | 0.051 | 0.046 | 0.062 | 0.002 | 0.168 | 0.137 | 0.029 | 0.079 | 0.008 | 0.000 | 0.031 |
|  | N | 115 | 115 | 114 | 114 | 115 | 115 | 115 | 114 | 115 | 115 | 115 | 114 |
| BDI Item 8 | rho | 0.091 | .219* | .199* | 0.130 | 0.141 | -0.007 | 0.108 | .225* | 0.097 | .198* | .287** | .203* |
|  | Sig. (2-tailed) | 0.333 | 0.019 | 0.034 | 0.167 | 0.134 | 0.941 | 0.251 | 0.016 | 0.303 | 0.034 | 0.002 | 0.030 |
|  | N | 115 | 115 | 114 | 114 | 115 | 115 | 115 | 114 | 115 | 115 | 115 | 114 |
| BDI Item 9 | rho | 0.042 | -0.001 | 0.090 | 0.003 | .210* | 0.015 | 0.056 | 0.152 | 0.049 | 0.129 | 0.177 | 0.117 |
|  | Sig. (2-tailed) | 0.652 | 0.988 | 0.343 | 0.973 | 0.025 | 0.873 | 0.553 | 0.107 | 0.600 | 0.169 | 0.059 | 0.216 |
|  | N | 115 | 115 | 114 | 114 | 115 | 115 | 115 | 114 | 115 | 115 | 115 | 114 |
| BDI Item 10 | rho | 0.040 | 0.123 | 0.164 | -0.036 | 0.035 | 0.070 | 0.154 | 0.152 | -0.033 | .219* | 0.077 | 0.090 |
|  | Sig. (2-tailed) | 0.670 | 0.193 | 0.082 | 0.707 | 0.712 | 0.457 | 0.103 | 0.108 | 0.725 | 0.019 | 0.418 | 0.342 |
|  | N | 114 | 114 | 113 | 113 | 114 | 114 | 114 | 113 | 114 | 114 | 114 | 113 |
| BDI Item 11 | rho | 0.168 | 0.078 | .248** | 0.163 | 0.067 | -0.156 | 0.121 | .205* | .187* | .184* | 0.115 | .235* |
|  | Sig. (2-tailed) | 0.072 | 0.409 | 0.008 | 0.084 | 0.475 | 0.097 | 0.197 | 0.029 | 0.046 | 0.049 | 0.222 | 0.012 |
|  | N | 115 | 115 | 114 | 114 | 115 | 115 | 115 | 114 | 115 | 115 | 115 | 114 |
| BDI Item 12 | rho | 0.100 | 0.166 | 0.056 | 0.063 | 0.038 | -0.085 | 0.105 | 0.160 | 0.020 | .203* | 0.109 | 0.162 |
|  | Sig. (2-tailed) | 0.286 | 0.075 | 0.552 | 0.509 | 0.683 | 0.369 | 0.264 | 0.090 | 0.833 | 0.029 | 0.246 | 0.085 |
|  | N | 115 | 115 | 114 | 114 | 115 | 115 | 115 | 114 | 115 | 115 | 115 | 114 |
| BDI Item 13 | rho | 0.114 | .206* | .254** | 0.162 | .241** | 0.033 | 0.154 | 0.183 | 0.128 | .282** | .240** | .210* |
|  | Sig. (2-tailed) | 0.225 | 0.027 | 0.006 | 0.084 | 0.009 | 0.726 | 0.101 | 0.051 | 0.174 | 0.002 | 0.010 | 0.025 |
|  | N | 115 | 115 | 114 | 114 | 115 | 115 | 115 | 114 | 115 | 115 | 115 | 114 |
| BDI Item 14 | rho | 0.139 | .230* | 0.148 | 0.098 | .190* | 0.019 | 0.102 | .252** | 0.094 | .264** | .238* | .227* |
|  | Sig. (2-tailed) | 0.139 | 0.013 | 0.115 | 0.298 | 0.042 | 0.841 | 0.278 | 0.007 | 0.319 | 0.004 | 0.010 | 0.015 |
|  | N | 115 | 115 | 114 | 114 | 115 | 115 | 115 | 114 | 115 | 115 | 115 | 114 |
| BDI Item 15 | rho | 0.088 | 0.099 | 0.039 | 0.090 | 0.073 | 0.035 | 0.034 | 0.136 | -0.006 | .230* | 0.049 | 0.126 |
|  | Sig. (2-tailed) | 0.353 | 0.292 | 0.685 | 0.343 | 0.442 | 0.715 | 0.722 | 0.152 | 0.946 | 0.014 | 0.607 | 0.184 |
|  | N | 114 | 114 | 113 | 113 | 114 | 114 | 114 | 113 | 114 | 114 | 114 | 113 |
| BDI Item 16 | rho | -0.085 | -0.077 | 0.014 | -0.086 | -0.046 | -0.164 | -0.052 | 0.031 | -0.168 | 0.096 | -.191* | -0.020 |
|  | Sig. (2-tailed) | 0.368 | 0.420 | 0.887 | 0.366 | 0.628 | 0.083 | 0.581 | 0.747 | 0.075 | 0.313 | 0.043 | 0.836 |
|  | N | 113 | 113 | 112 | 112 | 113 | 113 | 113 | 112 | 113 | 113 | 113 | 112 |
| BDI Item 17 | rho | -0.016 | 0.006 | .198* | 0.132 | .217* | -0.094 | 0.113 | .220* | 0.048 | 0.168 | 0.179 | .285** |
|  | Sig. (2-tailed) | 0.865 | 0.947 | 0.035 | 0.163 | 0.020 | 0.316 | 0.229 | 0.019 | 0.612 | 0.073 | 0.056 | 0.002 |
|  | N | 115 | 115 | 114 | 114 | 115 | 115 | 115 | 114 | 115 | 115 | 115 | 114 |
| BDI Item 18 | rho | 0.111 | 0.029 | 0.152 | 0.063 | .190* | 0.123 | -0.011 | 0.082 | 0.151 | .215* | 0.099 | 0.176 |
|  | Sig. (2-tailed) | 0.236 | 0.755 | 0.106 | 0.504 | 0.042 | 0.189 | 0.909 | 0.387 | 0.108 | 0.021 | 0.292 | 0.062 |
|  | N | 115 | 115 | 114 | 114 | 115 | 115 | 115 | 114 | 115 | 115 | 115 | 114 |
| BDI Item 19 | rho | .210* | 0.103 | .257** | 0.017 | -0.004 | -0.127 | .220* | 0.178 | 0.058 | 0.066 | 0.089 | 0.047 |
|  | Sig. (2-tailed) | 0.025 | 0.273 | 0.006 | 0.857 | 0.964 | 0.176 | 0.018 | 0.058 | 0.540 | 0.485 | 0.344 | 0.617 |
|  | N | 115 | 115 | 114 | 114 | 115 | 115 | 115 | 114 | 115 | 115 | 115 | 114 |
| BDI Item 20 | rho | 0.136 | 0.182 | 0.158 | 0.081 | 0.104 | 0.118 | 0.023 | .202* | 0.100 | .184* | 0.141 | 0.162 |
|  | Sig. (2-tailed) | 0.146 | 0.052 | 0.094 | 0.393 | 0.271 | 0.208 | 0.804 | 0.031 | 0.289 | 0.049 | 0.134 | 0.085 |
|  | N | 115 | 115 | 114 | 114 | 115 | 115 | 115 | 114 | 115 | 115 | 115 | 114 |
| BDI Item 21 | rho | 0.035 | 0.130 | 0.153 | 0.162 | 0.159 | 0.008 | 0.099 | 0.125 | -0.013 | .317** | 0.054 | .200* |
|  | Sig. (2-tailed) | 0.711 | 0.168 | 0.103 | 0.085 | 0.090 | 0.932 | 0.294 | 0.185 | 0.887 | 0.001 | 0.568 | 0.033 |
|  | N | 115 | 115 | 114 | 114 | 115 | 115 | 115 | 114 | 115 | 115 | 115 | 114 |
| ** p<0.01 two-tailed. | |  |  |  |  |  |  |  |  |  |  |  |  |
| * p<0.05 two-tailed. | |  |  |  |  |  |  |  |  |  |  |  |  |
| row: items of the IMP/EL subsclae | | |  |  |  |  |  |  |  |  |  |  |  |
| column: items of the BDI-II scale | | |  |  |  |  |  |  |  |  |  |  |  |

**Table S4. Results of independent sample t-tests for sex between clinical characterizes.**

| Independent sample t-test | | Levene's Test for Equality of Variances | | t-Test for Equality of Means | | | | | | | |
| --- | --- | --- | --- | --- | --- | --- | --- | --- | --- | --- | --- |
|  |  | F | Sig. | T | df | Significance | | Mean Difference | Std.Error Difference | 95% Confidence Intervall of the Difference | |
|  |  |  |  |  |  | sig. (1-tailed) | sig. (2-tailed) |  |  | Lower | Upper |
| Weight | equal variances are assumed | 0.248 | 0.620 | 5.869 | 113 | 0.000 | 0.000 | 18.182 | 3.098 | 12.044 | 24.319 |
|  | equal variances not assumed |  |  | 6.073 | 77.277 | 0.000 | 0.000 | 18.182 | 2.994 | 12.221 | 24.143 |
| Hight | equal variances are assumed | 2.414 | 0.123 | 10.913 | 113 | 0.000 | 0.000 | 15.838 | 1.451 | 12.963 | 18.713 |
|  | equal variances not assumed |  |  | 12.049 | 91.361 | 0.000 | 0.000 | 15.838 | 1.314 | 13.227 | 18.449 |
| BMI | equal variances are assumed | 0.605 | 0.438 | 1.506 | 113 | 0.067 | 0.135 | 1.396 | 0.927 | -0.440 | 3.232 |
|  | equal variances not assumed |  |  | 1.432 | 62.574 | 0.079 | 0.157 | 1.396 | 0.975 | -0.553 | 3.345 |
| BDI | equal variances are assumed | 8.633 | 0.004 | -1.672 | 113 | 0.049 | 0.097 | -3.553 | 2.125 | -7.763 | 0.658 |
|  | equal variances not assumed |  |  | -1.486 | 53.979 | 0.072 | 0.143 | -3.553 | 2.391 | -8.347 | 1.241 |
| CAARS-IA/ME | equal variances are assumed | 0.107 | 0.745 | -1.775 | 113 | 0.039 | 0.079 | -3.503 | 1.973 | -7.412 | 0.406 |
|  | equal variances not assumed |  |  | -1.810 | 74.351 | 0.037 | 0.074 | -3.503 | 1.935 | -7.359 | 0.353 |
| CAARS-IMP/EL | equal variances are assumed | 0.044 | 0.835 | -2.597 | 113 | 0.005 | 0.011 | -6.936 | 2.671 | -12.227 | -1.644 |
|  | equal variances not assumed |  |  | -2.654 | 74.830 | 0.005 | 0.010 | -6.936 | 2.614 | -12.142 | -1.729 |
| CAARS-HY/RE | equal variances are assumed | 1.168 | 0.282 | -2.118 | 113 | 0.018 | 0.036 | -5.303 | 2.504 | -10.264 | -.342 |
|  | equal variances not assumed |  |  | -2.048 | 65.192 | 0.022 | 0.045 | -5.303 | 2.589 | -10.474 | -.132 |
| CAARS-SC | equal variances are assumed | 2.072 | 0.153 | -1.202 | 113 | 0.116 | 0.232 | -3.223 | 2.681 | -8.534 | 2.088 |
|  | equal variances not assumed |  |  | -1.276 | 82.532 | 0.103 | 0.206 | -3.223 | 2.526 | -8.248 | 1.802 |
| CAARS-DSMIA | equal variances are assumed | 2.484 | 0.118 | -1.660 | 113 | 0.050 | 0.100 | -3.003 | 1.809 | -6.587 | .581 |
|  | equal variances not assumed |  |  | -1.794 | 86.548 | 0.038 | 0.076 | -3.003 | 1.674 | -6.330 | .324 |
| CAARS-DSMHYI | equal variances are assumed | 0.784 | 0.378 | -2.805 | 113 | 0.003 | 0.006 | -6.559 | 2.338 | -11.192 | -1.926 |
|  | equal variances not assumed |  |  | -2.734 | 66.427 | 0.004 | 0.008 | -6.559 | 2.399 | -11.349 | -1.769 |
| CAARS-G | equal variances are assumed | 1.634 | 0.204 | -2.166 | 113 | 0.016 | 0.032 | -3.927 | 1.813 | -7.518 | -.335 |
|  | equal variances not assumed |  |  | -2.274 | 80.222 | 0.013 | 0.026 | -3.927 | 1.727 | -7.363 | -.490 |
| CAARS-ADHD-Index | equal variances are assumed | 4.523 | 0.036 | -1.596 | 113 | 0.057 | 0.113 | -2.793 | 1.750 | -6.259 | .674 |
|  | equal variances not assumed |  |  | -1.757 | 90.651 | 0.041 | 0.082 | -2.793 | 1.590 | -5.951 | .365 |
| CGI-S | equal variances are assumed | 1.334 | 0.251 | -0.514 | 108 | 0.304 | 0.608 | -0.099 | 0.193 | -0.481 | 0.283 |
|  | equal variances not assumed |  |  | -0.583 | 91.598 | 0.281 | 0.561 | -0.099 | 0.170 | -0.436 | 0.238 |
| ADHSSB-G | equal variances are assumed | 3.135 | 0.079 | -1.653 | 113 | 0.051 | 0.101 | -3.005 | 1.818 | -6.606 | 0.596 |
|  | equal variances not assumed |  |  | -1.447 | 52.339 | 0.077 | 0.154 | -3.005 | 2.077 | -7.171 | 1.161 |
| ADHSSB-IA | equal variances are assumed | 2.828 | 0.095 | -1.026 | 113 | 0.154 | 0.307 | -0.868 | 0.847 | -2.545 | 0.809 |
|  | equal variances not assumed |  |  | -0.909 | 53.723 | 0.184 | 0.367 | -0.868 | 0.955 | -2.783 | 1.046 |
| ADHSSB-HY | equal variances are assumed | 1.490 | 0.225 | -0.245 | 113 | 0.403 | 0.807 | -0.168 | 0.684 | -1.524 | 1.188 |
|  | equal variances not assumed |  |  | -0.231 | 61.106 | 0.409 | 0.818 | -0.168 | 0.728 | -1.622 | 1.287 |
| ADHSSB-I | equal variances are assumed | 4.731 | 0.032 | -2.958 | 113 | 0.002 | 0.004 | -1.534 | 0.519 | -2.562 | -0.507 |
|  | equal variances not assumed |  |  | -2.687 | 56.521 | 0.005 | 0.009 | -1.534 | 0.571 | -2.678 | -0.390 |
| ASRS-A | equal variances are assumed | 0.678 | 0.412 | -0.190 | 111 | 0.425 | 0.849 | -0.034 | 0.176 | -0.383 | 0.316 |
|  | equal variances not assumed |  |  | -0.198 | 76.035 | 0.422 | 0.843 | -0.034 | 0.169 | -0.371 | 0.304 |
| ASRS-B | equal variances are assumed | 1.475 | 0.227 | -1.857 | 111 | 0.033 | 0.066 | -0.842 | 0.454 | -1.741 | 0.056 |
|  | equal variances not assumed |  |  | -1.941 | 73.031 | 0.028 | 0.056 | -0.842 | 0.434 | -1.707 | 0.022 |
| WURS-K | equal variances are assumed | 0.000 | 0.999 | -0.351 | 110 | 0.363 | 0.726 | -0.814 | 2.317 | -5.406 | 3.777 |
|  | equal variances not assumed |  |  | -0.355 | 70.561 | 0.362 | 0.724 | -0.814 | 2.294 | -5.389 | 3.760 |
| WHOQOL-BREF | equal variances are assumed | 2.516 | 0.115 | 1.378 | 113 | 0.085 | 0.171 | 13.134 | 9.531 | -5.749 | 32.017 |
|  | equal variances not assumed |  |  | 1.298 | 61.253 | 0.100 | 0.199 | 13.134 | 10.120 | -7.101 | 33.369 |
| physical health | equal variances are assumed | 0.720 | 0.398 | 1.681 | 113 | 0.048 | 0.095 | 4.721 | 2.808 | -0.843 | 10.284 |
|  | equal variances not assumed |  |  | 1.560 | 59.241 | 0.062 | 0.124 | 4.721 | 3.026 | -1.333 | 10.775 |
| psychological health | equal variances are assumed | 3.998 | 0.048 | 1.039 | 113 | 0.151 | 0.301 | 3.384 | 3.257 | -3.069 | 9.837 |
|  | equal variances not assumed |  |  | 0.946 | 56.747 | 0.174 | 0.348 | 3.384 | 3.579 | -3.784 | 10.552 |
| social relationship | equal variances are assumed | 0.065 | 0.799 | 0.295 | 113 | 0.384 | 0.769 | 1.146 | 3.888 | -6.556 | 8.849 |
|  | equal variances not assumed |  |  | 0.292 | 68.853 | 0.386 | 0.771 | 1.146 | 3.932 | -6.697 | 8.990 |
| environment | variances are equal | 5.325 | 0.023 | 1.361 | 113 | 0.088 | 0.176 | 3.884 | 2.854 | -1.770 | 9.538 |
|  | variances are not equal |  |  | 1.224 | 55.378 | 0.113 | 0.226 | 3.884 | 3.172 | -2.472 | 10.239 |
| IIP | variances are equal | 3.423 | 0.067 | -1.997 | 113 | 0.024 | 0.048 | -13.338 | 6.680 | -26.573 | -0.103 |
|  | variances are not equal |  |  | -2.239 | 94.942 | 0.014 | 0.027 | -13.338 | 5.956 | -25.163 | -1.513 |

**Table S5**. Comparisons of baseline clinical characteristics among groups ED-. ED+ without and ED+ with comorbid depressive symptoms

| **ANOVA** | | | | | | |
| --- | --- | --- | --- | --- | --- | --- |
|  | | Sum of Squares | df | Mean Square | F | Sig. |
| WURS-K (sum) | Between Groups | 1355.675 | 2 | 677.837 | 5.646 | .005 |
|  | Within Groups | 13086.602 | 109 | 120.061 |  |  |
|  | Total | 14442.277 | 111 |  |  |  |
| CAARS (T-score ) IA/ME | Between Groups | 1735.547 | 2 | 867.774 | 10.112 | <.001 |
|  | Within Groups | 9611.548 | 112 | 85.817 |  |  |
|  | Total | 11347.096 | 114 |  |  |  |
| CAARS (T-score ) IMP/EL | Between Groups | 14883.995 | 2 | 7441.997 | 127.164 | <.001 |
|  | Within Groups | 6554.579 | 112 | 58.523 |  |  |
|  | Total | 21438.574 | 114 |  |  |  |
| CAARS (T-score ) HY/RE | Between Groups | 1744.172 | 2 | 872.086 | 5.833 | .004 |
|  | Within Groups | 16743.619 | 112 | 149.497 |  |  |
|  | Total | 18487.791 | 114 |  |  |  |
| CAARS (T-score ) SC | Between Groups | 1845.080 | 2 | 922.540 | 5.497 | .005 |
|  | Within Groups | 18796.781 | 112 | 167.828 |  |  |
|  | Total | 20641.861 | 114 |  |  |  |
| CAARS (T-score ) DSM-IA | Between Groups | 898.447 | 2 | 449.223 | 5.845 | .004 |
|  | Within Groups | 8607.675 | 112 | 76.854 |  |  |
|  | Total | 9506.122 | 114 |  |  |  |
| CAARS (T-score ) DSM-HYI | Between Groups | 4741.006 | 2 | 2370.503 | 22.413 | <.001 |
|  | Within Groups | 11845.776 | 112 | 105.766 |  |  |
|  | Total | 16586.783 | 114 |  |  |  |
| CAARS (T-score ) DSM-G | Between Groups | 2614.214 | 2 | 1307.107 | 20.637 | <.001 |
|  | Within Groups | 7093.734 | 112 | 63.337 |  |  |
|  | Total | 9707.948 | 114 |  |  |  |
| CAARS (T-score ) ADHS-Index | Between Groups | 3143.411 | 2 | 1571.706 | 30.695 | <.001 |
|  | Within Groups | 5734.850 | 112 | 51.204 |  |  |
|  | Total | 8878.261 | 114 |  |  |  |
| ADHD-SB (sum) IA | Between Groups | 72.860 | 2 | 36.430 | 2.062 | .132 |
|  | Within Groups | 1978.323 | 112 | 17.664 |  |  |
|  | Total | 2051.183 | 114 |  |  |  |
| ADHD-SB (sum) HY | Between Groups | 97.943 | 2 | 48.972 | 4.455 | .014 |
|  | Within Groups | 1231.239 | 112 | 10.993 |  |  |
|  | Total | 1329.183 | 114 |  |  |  |
| ADHD-SB (sum) I | Between Groups | 140.700 | 2 | 70.350 | 11.560 | <.001 |
|  | Within Groups | 681.596 | 112 | 6.086 |  |  |
|  | Total | 822.296 | 114 |  |  |  |
| ADHD-SB (sum) G | Between Groups | 1204.083 | 2 | 602.041 | 8.037 | <.001 |
|  | Within Groups | 8390.300 | 112 | 74.913 |  |  |
|  | Total | 9594.383 | 114 |  |  |  |
| ASRS (sum) part A | Between Groups | 4.049 | 2 | 2.025 | 2.761 | .068 |
|  | Within Groups | 80.676 | 110 | .733 |  |  |
|  | Total | 84.726 | 112 |  |  |  |
| ASRS (sum) part B | Between Groups | 85.362 | 2 | 42.681 | 9.707 | <.001 |
|  | Within Groups | 483.647 | 110 | 4.397 |  |  |
|  | Total | 569.009 | 112 |  |  |  |
| BDI (sum) | Between Groups | 7684.043 | 2 | 3842.021 | 79.113 | <.001 |
|  | Within Groups | 5439.122 | 112 | 48.564 |  |  |
|  | Total | 13123.165 | 114 |  |  |  |
| CGI-S (sum) | Between Groups | 2.740 | 2 | 1.370 | 1.572 | .212 |
|  | Within Groups | 93.224 | 107 | .871 |  |  |
|  | Total | 95.964 | 109 |  |  |  |
| IIP (sum) | Between Groups | 31277.988 | 2 | 15638.994 | 17.562 | <.001 |
|  | Within Groups | 99738.499 | 112 | 890.522 |  |  |
|  | Total | 131016.487 | 114 |  |  |  |
| WHOQOL-BREF (Sum) | Between Groups | 72312.635 | 2 | 36156.318 | 21.354 | <.001 |
|  | Within Groups | 189636.839 | 112 | 1693.186 |  |  |
|  | Total | 261949.474 | 114 |  |  |  |
| WHOQOL-BREF Physical health | Between Groups | 2691.158 | 2 | 1345.579 | 7.449 | <.001 |
|  | Within Groups | 20230.609 | 112 | 180.630 |  |  |
|  | Total | 22921.767 | 114 |  |  |  |
| WHOQOL-BREF Psychological health | Between Groups | 7813.777 | 2 | 3906.888 | 19.395 | <.001 |
|  | Within Groups | 22561.313 | 112 | 201.440 |  |  |
|  | Total | 30375.090 | 114 |  |  |  |
| WHOQOL-BREF Social relationship | Between Groups | 4056.455 | 2 | 2028.227 | 5.848 | .004 |
|  | Within Groups | 38843.328 | 112 | 346.815 |  |  |
|  | Total | 42899.782 | 114 |  |  |  |
| WHOQOL-BREF Environment | Between Groups | 4736.794 | 2 | 2368.397 | 14.158 | <.001 |
|  | Within Groups | 18735.653 | 112 | 167.283 |  |  |
|  | Total | 23472.447 | 114 |  |  |  |

| **Multiple comparisons** | | | | | | | |
| --- | --- | --- | --- | --- | --- | --- | --- |
| Bonferroni | | | | | | | |
| Dependent Variable | (I) | (J) | Mean Difference z (I-J) | Std. Error | Sig. | 95% Confidence Interval | |
|  |  |  |  |  |  | Lower Bound | Upper Bound |
| WURS-K (sum) | ED+ Dep- | ED+ Dep+ | -1.180 | 2.955 | 1.000 | -8.37 | 6.01 |
|  |  | ED- | 6.331 | 2.529 | .041 | .18 | 12.48 |
|  | ED+ Dep+ | ED+ Dep- | 1.180 | 2.955 | 1.000 | -6.01 | 8.37 |
|  |  | ED- | 7.511 | 2.560 | .012 | 1.29 | 13.74 |
|  | ED- | ED+ Dep- | -6.331 | 2.529 | .041 | -12.48 | -.18 |
|  |  | ED+ Dep+ | -7.511 | 2.560 | .012 | -13.74 | -1.29 |
| CAARS (T-score ) IA/ME | ED+ Dep- | ED+ Dep+ | .561 | 2.477 | 1.000 | -5.46 | 6.58 |
|  |  | ED- | 8.033 | 2.101 | <.001 | 2.93 | 13.14 |
|  | ED+ Dep+ | ED+ Dep- | -.561 | 2.477 | 1.000 | -6.58 | 5.46 |
|  |  | ED- | 7.472 | 2.152 | .002 | 2.24 | 12.70 |
|  | ED- | ED+ Dep- | -8.033 | 2.101 | <.001 | -13.14 | -2.93 |
|  |  | ED+ Dep+ | -7.472 | 2.152 | .002 | -12.70 | -2.24 |
| CAARS (T-score ) IMP/EL | ED+ Dep- | ED+ Dep+ | -1.909 | 2.046 | 1.000 | -6.88 | 3.06 |
|  |  | ED- | 21.801 | 1.735 | <.001 | 17.58 | 26.02 |
|  | ED+ Dep+ | ED+ Dep- | 1.909 | 2.046 | 1.000 | -3.06 | 6.88 |
|  |  | ED- | 23.711 | 1.777 | <.001 | 19.39 | 28.03 |
|  | ED- | ED+ Dep- | -21.801 | 1.735 | <.001 | -26.02 | -17.58 |
|  |  | ED+ Dep+ | -23.711 | 1.777 | <.001 | -28.03 | -19.39 |
| CAARS (T-score ) HY/RE | ED+ Dep- | ED+ Dep+ | -5.421 | 3.270 | .300 | -13.37 | 2.53 |
|  |  | ED- | 4.198 | 2.773 | .399 | -2.54 | 10.94 |
|  | ED+ Dep+ | ED+ Dep- | 5.421 | 3.270 | .300 | -2.53 | 13.37 |
|  |  | ED- | 9.620 | 2.841 | .003 | 2.71 | 16.52 |
|  | ED- | ED+ Dep- | -4.198 | 2.773 | .399 | -10.94 | 2.54 |
|  |  | ED+ Dep+ | -9.620 | 2.841 | .003 | -16.52 | -2.71 |
| CAARS (T-score ) SC | ED+ Dep- | ED+ Dep+ | -1.305 | 3.465 | 1.000 | -9.73 | 7.12 |
|  |  | ED- | 7.333 | 2.938 | .042 | .19 | 14.47 |
|  | ED+ Dep+ | ED+ Dep- | 1.305 | 3.465 | 1.000 | -7.12 | 9.73 |
|  |  | ED- | 8.638 | 3.010 | .015 | 1.32 | 15.95 |
|  | ED- | ED+ Dep- | -7.333 | 2.938 | .042 | -14.47 | -.19 |
|  |  | ED+ Dep+ | -8.638 | 3.010 | .015 | -15.95 | -1.32 |
| CAARS (T-score ) DSM-IA | ED+ Dep- | ED+ Dep+ | -.046 | 2.344 | 1.000 | -5.74 | 5.65 |
|  |  | ED- | 5.570 | 1.988 | .018 | .74 | 10.40 |
|  | ED+ Dep+ | ED+ Dep- | .046 | 2.344 | 1.000 | -5.65 | 5.74 |
|  |  | ED- | 5.616 | 2.037 | .020 | .67 | 10.57 |
|  | ED- | ED+ Dep- | -5.570 | 1.988 | .018 | -10.40 | -.74 |
|  |  | ED+ Dep+ | -5.616 | 2.037 | .020 | -10.57 | -.67 |
| CAARS (T-score ) DSM-HYI | ED+ Dep- | ED+ Dep+ | -4.388 | 2.750 | .340 | -11.07 | 2.30 |
|  |  | ED- | 10.360 | 2.332 | <.001 | 4.69 | 16.03 |
|  | ED+ Dep+ | ED+ Dep- | 4.388 | 2.750 | .340 | -2.30 | 11.07 |
|  |  | ED- | 14.748 | 2.390 | <.001 | 8.94 | 20.56 |
|  | ED- | ED+ Dep- | -10.360 | 2.332 | <.001 | -16.03 | -4.69 |
|  |  | ED+ Dep+ | -14.748 | 2.390 | <.001 | -20.56 | -8.94 |
| CAARS (T-score ) DSM-G | ED+ Dep- | ED+ Dep+ | -1.521 | 2.128 | 1.000 | -6.69 | 3.65 |
|  |  | ED- | 8.746 | 1.805 | <.001 | 4.36 | 13.13 |
|  | ED+ Dep+ | ED+ Dep- | 1.521 | 2.128 | 1.000 | -3.65 | 6.69 |
|  |  | ED- | 10.267 | 1.849 | <.001 | 5.77 | 14.76 |
|  | ED- | ED+ Dep- | -8.746 | 1.805 | <.001 | -13.13 | -4.36 |
|  |  | ED+ Dep+ | -10.267 | 1.849 | <.001 | -14.76 | -5.77 |
| CAARS (T-score ) ADHS-Index | ED+ Dep- | ED+ Dep+ | -2.172 | 1.914 | .776 | -6.82 | 2.48 |
|  |  | ED- | 9.302 | 1.623 | <.001 | 5.36 | 13.25 |
|  | ED+ Dep+ | ED+ Dep- | 2.172 | 1.914 | .776 | -2.48 | 6.82 |
|  |  | ED- | 11.475 | 1.663 | <.001 | 7.43 | 15.52 |
|  | ED- | ED+ Dep- | -9.302 | 1.623 | <.001 | -13.25 | -5.36 |
|  |  | ED+ Dep+ | -11.475 | 1.663 | <.001 | -15.52 | -7.43 |
| ADHD-SB (sum) IA | ED+ Dep- | ED+ Dep+ | -1.289 | 1.124 | .762 | -4.02 | 1.44 |
|  |  | ED- | .693 | .953 | 1.000 | -1.62 | 3.01 |
|  | ED+ Dep+ | ED+ Dep- | 1.289 | 1.124 | .762 | -1.44 | 4.02 |
|  |  | ED- | 1.982 | .977 | .134 | -.39 | 4.36 |
|  | ED- | ED+ Dep- | -.693 | .953 | 1.000 | -3.01 | 1.62 |
|  |  | ED+ Dep+ | -1.982 | .977 | .134 | -4.36 | .39 |
| ADHD-SB (sum) HY | ED+ Dep- | ED+ Dep+ | -2.022 | .887 | .073 | -4.18 | .13 |
|  |  | ED- | .217 | .752 | 1.000 | -1.61 | 2.04 |
|  | ED+ Dep+ | ED+ Dep- | 2.022 | .887 | .073 | -.13 | 4.18 |
|  |  | ED- | 2.239 | .770 | .013 | .37 | 4.11 |
|  | ED- | ED+ Dep- | -.217 | .752 | 1.000 | -2.04 | 1.61 |
|  |  | ED+ Dep+ | -2.239 | .770 | .013 | -4.11 | -.37 |
| ADHD-SB (sum) I | ED+ Dep- | ED+ Dep+ | -2.091 | .660 | .006 | -3.69 | -.49 |
|  |  | ED- | .656 | .559 | .730 | -.70 | 2.02 |
|  | ED+ Dep+ | ED+ Dep- | 2.091 | .660 | .006 | .49 | 3.69 |
|  |  | ED- | 2.747 | .573 | <.001 | 1.35 | 4.14 |
|  | ED- | ED+ Dep- | -.656 | .559 | .730 | -2.02 | .70 |
|  |  | ED+ Dep+ | -2.747 | .573 | <.001 | -4.14 | -1.35 |
| ADHD-SB (sum) G | ED+ Dep- | ED+ Dep+ | -5.944 | 2.315 | .035 | -11.57 | -.32 |
|  |  | ED- | 2.106 | 1.963 | .857 | -2.66 | 6.88 |
|  | ED+ Dep+ | ED+ Dep- | 5.944 | 2.315 | .035 | .32 | 11.57 |
|  |  | ED- | 8.050 | 2.011 | <.001 | 3.16 | 12.94 |
|  | ED- | ED+ Dep- | -2.106 | 1.963 | .857 | -6.88 | 2.66 |
|  |  | ED+ Dep+ | -8.050 | 2.011 | <.001 | -12.94 | -3.16 |
| ASRS (sum) part A | ED+ Dep- | ED+ Dep+ | -.360 | .229 | .356 | -.92 | .20 |
|  |  | ED- | .108 | .195 | 1.000 | -.37 | .58 |
|  | ED+ Dep+ | ED+ Dep- | .360 | .229 | .356 | -.20 | .92 |
|  |  | ED- | .468 | .200 | .064 | -.02 | .95 |
|  | ED- | ED+ Dep- | -.108 | .195 | 1.000 | -.58 | .37 |
|  |  | ED+ Dep+ | -.468 | .200 | .064 | -.95 | .02 |
| ASRS (sum) part B | ED+ Dep- | ED+ Dep+ | -.784 | .561 | .494 | -2.15 | .58 |
|  |  | ED- | 1.270 | .478 | .027 | .11 | 2.43 |
|  | ED+ Dep+ | ED+ Dep- | .784 | .561 | .494 | -.58 | 2.15 |
|  |  | ED- | 2.055 | .490 | <.001 | .86 | 3.25 |
|  | ED- | ED+ Dep- | -1.270 | .478 | .027 | -2.43 | -.11 |
|  |  | ED+ Dep+ | -2.055 | .490 | <.001 | -3.25 | -.86 |
| BDI (sum) | ED+ Dep- | ED+ Dep+ | -21.960 | 1.864 | <.001 | -26.49 | -17.43 |
|  |  | ED- | -4.865 | 1.580 | .008 | -8.71 | -1.02 |
|  | ED+ Dep+ | ED+ Dep- | 21.960 | 1.864 | <.001 | 17.43 | 26.49 |
|  |  | ED- | 17.095 | 1.619 | <.001 | 13.16 | 21.03 |
|  | ED- | ED+ Dep- | 4.865 | 1.580 | .008 | 1.02 | 8.71 |
|  |  | ED+ Dep+ | -17.095 | 1.619 | <.001 | -21.03 | -13.16 |
| CGI-S (sum) | ED+ Dep- | ED+ Dep+ | -.173 | .252 | 1.000 | -.79 | .44 |
|  |  | ED- | .206 | .217 | 1.000 | -.32 | .73 |
|  | ED+ Dep+ | ED+ Dep- | .173 | .252 | 1.000 | -.44 | .79 |
|  |  | ED- | .379 | .219 | .260 | -.15 | .91 |
|  | ED- | ED+ Dep- | -.206 | .217 | 1.000 | -.73 | .32 |
|  |  | ED+ Dep+ | -.379 | .219 | .260 | -.91 | .15 |
| IIP (sum) | ED+ Dep- | ED+ Dep+ | -24.462 | 7.981 | .008 | -43.86 | -5.07 |
|  |  | ED- | 16.445 | 6.768 | .050 | .00 | 32.89 |
|  | ED+ Dep+ | ED+ Dep- | 24.462 | 7.981 | .008 | 5.07 | 43.86 |
|  |  | ED- | 40.907 | 6.934 | <.001 | 24.05 | 57.76 |
|  | ED- | ED+ Dep- | -16.445 | 6.768 | .050 | -32.89 | .00 |
|  |  | ED+ Dep+ | -40.907 | 6.934 | <.001 | -57.76 | -24.05 |
| WHOQOL-BREF (Sum) | ED+ Dep- | ED+ Dep+ | 57.41727 | 11.00438 | <.001 | 30.6715 | 84.1631 |
|  |  | ED- | -2.52463 | 9.33187 | 1.000 | -25.2055 | 20.1562 |
|  | ED+ Dep+ | ED+ Dep- | -57.41727 | 11.00438 | <.001 | -84.1631 | -30.6715 |
|  |  | ED- | -59.94190 | 9.56079 | <.001 | -83.1791 | -36.7047 |
|  | ED- | ED+ Dep- | 2.52463 | 9.33187 | 1.000 | -20.1562 | 25.2055 |
|  |  | ED+ Dep+ | 59.94190 | 9.56079 | <.001 | 36.7047 | 83.1791 |
| WHOQOL-BREF Physical health | ED+ Dep- | ED+ Dep+ | 12.32909 | 3.59425 | .003 | 3.5934 | 21.0648 |
|  |  | ED- | 1.50673 | 3.04798 | 1.000 | -5.9013 | 8.9147 |
|  | ED+ Dep+ | ED+ Dep- | -12.32909 | 3.59425 | .003 | -21.0648 | -3.5934 |
|  |  | ED- | -10.82236 | 3.12275 | .002 | -18.4121 | -3.2326 |
|  | ED- | ED+ Dep- | -1.50673 | 3.04798 | 1.000 | -8.9147 | 5.9013 |
|  |  | ED+ Dep+ | 10.82236 | 3.12275 | .002 | 3.2326 | 18.4121 |
| WHOQOL-BREF Psychological health | ED+ Dep- | ED+ Dep+ | 20.21686 | 3.79565 | <.001 | 10.9916 | 29.4421 |
|  |  | ED- | 1.20004 | 3.21877 | 1.000 | -6.6231 | 9.0232 |
|  | ED+ Dep+ | ED+ Dep- | -20.21686 | 3.79565 | <.001 | -29.4421 | -10.9916 |
|  |  | ED- | -19.01682 | 3.29773 | <.001 | -27.0318 | -11.0018 |
|  | ED- | ED+ Dep- | -1.20004 | 3.21877 | 1.000 | -9.0232 | 6.6231 |
|  |  | ED+ Dep+ | 19.01682 | 3.29773 | <.001 | 11.0018 | 27.0318 |
| WHOQOL-BREF Social relationship | ED+ Dep- | ED+ Dep+ | 10.08995 | 4.98038 | .135 | -2.0147 | 22.1946 |
|  |  | ED- | -4.70829 | 4.22343 | .802 | -14.9732 | 5.5566 |
|  | ED+ Dep+ | ED+ Dep- | -10.08995 | 4.98038 | .135 | -22.1946 | 2.0147 |
|  |  | ED- | -14.79824 | 4.32704 | .003 | -25.3150 | -4.2815 |
|  | ED- | ED+ Dep- | 4.70829 | 4.22343 | .802 | -5.5566 | 14.9732 |
|  |  | ED+ Dep+ | 14.79824 | 4.32704 | .003 | 4.2815 | 25.3150 |
| WHOQOL-BREF Environment | ED+ Dep- | ED+ Dep+ | 14.78238 | 3.45890 | <.001 | 6.3756 | 23.1891 |
|  |  | ED- | -.52292 | 2.93320 | 1.000 | -7.6520 | 6.6061 |
|  | ED+ Dep+ | ED+ Dep- | -14.78238 | 3.45890 | <.001 | -23.1891 | -6.3756 |
|  |  | ED- | -15.30529 | 3.00515 | <.001 | -22.6092 | -8.0014 |
|  | ED- | ED+ Dep- | .52292 | 2.93320 | 1.000 | -6.6061 | 7.6520 |
|  |  | ED+ Dep+ | 15.30529 | 3.00515 | <.001 | 8.0014 | 22.6092 |
| * The mean difference is significant at the 0.05 level.  ED- means ADHD participants without emotional dysregulation; ED+ means ADHD participants with emotional dysregulation  Dep- means ADHD participants without comorbid depressive symptoms; Dep+ means ADHD participants with comorbid depressive symptoms | | | | | | | |

**Table S6**. Comparisons of baseline EEG characteristics among groups ED-. ED+ without and ED+ with comorbid depressive symptoms

| **ANOVA** | | | | | | |
| --- | --- | --- | --- | --- | --- | --- |
|  | | Sum of Squares | df | Mean Square | F | Sig. |
| EEG-vigilance stages 0 (%) | Between Groups | 270.247 | 2 | 135.123 | .366 | .695 |
|  | Within Groups | 39534.330 | 107 | 369.480 |  |  |
|  | Total | 39804.577 | 109 |  |  |  |
| EEG-vigilance stages A1 (%) | Between Groups | 4649.378 | 2 | 2324.689 | 2.443 | .092 |
|  | Within Groups | 101800.762 | 107 | 951.409 |  |  |
|  | Total | 106450.140 | 109 |  |  |  |
| EEG-vigilance stages A2 (%) | Between Groups | 26.077 | 2 | 13.038 | .200 | .819 |
|  | Within Groups | 6980.709 | 107 | 65.240 |  |  |
|  | Total | 7006.786 | 109 |  |  |  |
| EEG-vigilance stages A3 (%) | Between Groups | 138.205 | 2 | 69.103 | 1.612 | .204 |
|  | Within Groups | 4586.454 | 107 | 42.864 |  |  |
|  | Total | 4724.659 | 109 |  |  |  |
| EEG-vigilance stages B1 (%) | Between Groups | 614.545 | 2 | 307.273 | .826 | .441 |
|  | Within Groups | 39807.525 | 107 | 372.033 |  |  |
|  | Total | 40422.071 | 109 |  |  |  |
| EEG-vigilance stages B2/3 (%) | Between Groups | 1235.288 | 2 | 617.644 | 2.713 | .071 |
|  | Within Groups | 24357.955 | 107 | 227.644 |  |  |
|  | Total | 25593.243 | 109 |  |  |  |
| EEG-vigilance stages C (%) | Between Groups | 654.656 | 2 | 327.328 | 2.177 | .118 |
|  | Within Groups | 16090.966 | 107 | 150.383 |  |  |
|  | Total | 16745.622 | 109 |  |  |  |
| Arousal stability score | Between Groups | 87.743 | 2 | 43.872 | 3.169 | .046 |
|  | Within Groups | 1481.211 | 107 | 13.843 |  |  |
|  | Total | 1568.955 | 109 |  |  |  |
| Mean EEG-vigilance level | Between Groups | 9.092 | 2 | 4.546 | 2.980 | .055 |
|  | Within Groups | 163.252 | 107 | 1.526 |  |  |
|  | Total | 172.344 | 109 |  |  |  |

| **Multiple comparisons** | | | | | | | |  |
| --- | --- | --- | --- | --- | --- | --- | --- | --- |
| Bonferroni | | | | | | | |  |
| Dependent Variable | (I) | (J) | Mean Difference z (I-J) | Std. Error | Sig. | 95% Confidence Interval | |  |
|  |  |  |  |  |  | Lower Bound | Upper Bound |  |
| EEG-vigilance stages 0 (%) | ED+ Dep- | ED+ Dep+ | -1.64192 | 5.38423 | 1.000 | -14.7375 | 11.4536 |  |
|  |  | ED- | 2.09949 | 4.58711 | 1.000 | -9.0573 | 13.2563 |  |
|  | ED+ Dep+ | ED+ Dep- | 1.64192 | 5.38423 | 1.000 | -11.4536 | 14.7375 |  |
|  |  | ED- | 3.74141 | 4.52472 | 1.000 | -7.2636 | 14.7464 |  |
|  | ED- | ED+ Dep- | -2.09949 | 4.58711 | 1.000 | -13.2563 | 9.0573 |  |
|  |  | ED+ Dep+ | -3.74141 | 4.52472 | 1.000 | -14.7464 | 7.2636 |  |
| EEG-vigilance stages A1 (%) | ED+ Dep- | ED+ Dep+ | -18.19546 | 8.63997 | .113 | -39.2096 | 2.8187 |  |
|  |  | ED- | -13.23997 | 7.36084 | .225 | -31.1430 | 4.6631 |  |
|  | ED+ Dep+ | ED+ Dep- | 18.19546 | 8.63997 | .113 | -2.8187 | 39.2096 |  |
|  |  | ED- | 4.95550 | 7.26073 | 1.000 | -12.7041 | 22.6151 |  |
|  | ED- | ED+ Dep- | 13.23997 | 7.36084 | .225 | -4.6631 | 31.1430 |  |
|  |  | ED+ Dep+ | -4.95550 | 7.26073 | 1.000 | -22.6151 | 12.7041 |  |
| EEG-vigilance stages A2 (%) | ED+ Dep- | ED+ Dep+ | -1.02337 | 2.26249 | 1.000 | -6.5262 | 4.4795 |  |
|  |  | ED- | -1.20387 | 1.92753 | 1.000 | -5.8920 | 3.4843 |  |
|  | ED+ Dep+ | ED+ Dep- | 1.02337 | 2.26249 | 1.000 | -4.4795 | 6.5262 |  |
|  |  | ED- | -.18050 | 1.90132 | 1.000 | -4.8049 | 4.4439 |  |
|  | ED- | ED+ Dep- | 1.20387 | 1.92753 | 1.000 | -3.4843 | 5.8920 |  |
|  |  | ED+ Dep+ | .18050 | 1.90132 | 1.000 | -4.4439 | 4.8049 |  |
| EEG-vigilance stages A3 (%) | ED+ Dep- | ED+ Dep+ | -.06391 | 1.83390 | 1.000 | -4.5243 | 4.3965 |  |
|  |  | ED- | -2.27991 | 1.56239 | .442 | -6.0800 | 1.5201 |  |
|  | ED+ Dep+ | ED+ Dep- | .06391 | 1.83390 | 1.000 | -4.3965 | 4.5243 |  |
|  |  | ED- | -2.21600 | 1.54114 | .460 | -5.9644 | 1.5324 |  |
|  | ED- | ED+ Dep- | 2.27991 | 1.56239 | .442 | -1.5201 | 6.0800 |  |
|  |  | ED+ Dep+ | 2.21600 | 1.54114 | .460 | -1.5324 | 5.9644 |  |
| EEG-vigilance stages B1 (%) | ED+ Dep- | ED+ Dep+ | 6.20612 | 5.40280 | .760 | -6.9346 | 19.3468 |  |
|  |  | ED- | 5.29005 | 4.60293 | .759 | -5.9052 | 16.4853 |  |
|  | ED+ Dep+ | ED+ Dep- | -6.20612 | 5.40280 | .760 | -19.3468 | 6.9346 |  |
|  |  | ED- | -.91608 | 4.54033 | 1.000 | -11.9591 | 10.1269 |  |
|  | ED- | ED+ Dep- | -5.29005 | 4.60293 | .759 | -16.4853 | 5.9052 |  |
|  |  | ED+ Dep+ | .91608 | 4.54033 | 1.000 | -10.1269 | 11.9591 |  |
| EEG-vigilance stages B2/3 (%) | ED+ Dep- | ED+ Dep+ | 8.14389 | 4.22627 | .170 | -2.1352 | 18.4230 |  |
|  |  | ED- | 7.92764 | 3.60058 | .089 | -.8297 | 16.6850 |  |
|  | ED+ Dep+ | ED+ Dep- | -8.14389 | 4.22627 | .170 | -18.4230 | 2.1352 |  |
|  |  | ED- | -.21625 | 3.55161 | 1.000 | -8.8545 | 8.4220 |  |
|  | ED- | ED+ Dep- | -7.92764 | 3.60058 | .089 | -16.6850 | .8297 |  |
|  |  | ED+ Dep+ | .21625 | 3.55161 | 1.000 | -8.4220 | 8.8545 |  |
| EEG-vigilance stages C (%) | ED+ Dep- | ED+ Dep+ | 6.57538 | 3.43501 | .175 | -1.7792 | 14.9300 |  |
|  |  | ED- | 1.40593 | 2.92646 | 1.000 | -5.7118 | 8.5237 |  |
|  | ED+ Dep+ | ED+ Dep- | -6.57538 | 3.43501 | .175 | -14.9300 | 1.7792 |  |
|  |  | ED- | -5.16945 | 2.88666 | .228 | -12.1904 | 1.8515 |  |
|  | ED- | ED+ Dep- | -1.40593 | 2.92646 | 1.000 | -8.5237 | 5.7118 |  |
|  |  | ED+ Dep+ | 5.16945 | 2.88666 | .228 | -1.8515 | 12.1904 |  |
| Arousal stability score | ED+ Dep- | ED+ Dep+ | -2.622 | 1.042 | .040 | -5.16 | -.09 |  |
|  |  | ED- | -1.262 | .888 | .475 | -3.42 | .90 |  |
|  | ED+ Dep+ | ED+ Dep- | 2.622 | 1.042 | .040 | .09 | 5.16 |  |
|  |  | ED- | 1.360 | .876 | .370 | -.77 | 3.49 |  |
|  | ED- | ED+ Dep- | 1.262 | .888 | .475 | -.90 | 3.42 |  |
|  |  | ED+ Dep+ | -1.360 | .876 | .370 | -3.49 | .77 |  |
| Mean EEG-vigilance level | ED+ Dep- | ED+ Dep+ | -.84296 | .34599 | .049 | -1.6845 | -.0014 |  |
|  |  | ED- | -.46577 | .29477 | .351 | -1.1827 | .2512 |  |
|  | ED+ Dep+ | ED+ Dep- | .84296 | .34599 | .049 | .0014 | 1.6845 |  |
|  |  | ED- | .37719 | .29076 | .592 | -.3300 | 1.0844 |  |
|  | ED- | ED+ Dep- | .46577 | .29477 | .351 | -.2512 | 1.1827 |  |
|  |  | ED+ Dep+ | -.37719 | .29076 | .592 | -1.0844 | .3300 |  |
| * The mean difference is significant at the 0.05 level.  ED- means ADHD participants without emotional dysregulation; ED+ means ADHD participants with emotional dysregulation  Dep- means ADHD participants without comorbid depressive symptoms; Dep+ means ADHD participants with comorbid depressive symptoms | | | | | | | | |
